# Supplementary material for: A genetic-epigenetic interplay at 1q21.1 locus underlies CHD1L-mediated vulnerability to primary progressive multiple sclerosis
Source: Nat Commun. 2024 Jul 30;15:6419. doi: 10.1038/s41467-024-50794-z (PMC11289459; doi:10.1038/s41467-024-50794-z)
Supplement: Supplementary file 1 — Supplementary Information [file 41467_2024_50794_MOESM1_ESM.pdf]

A genetic-epigenetic interplay at 1q21.1 locus underlies *CHD1L*-mediated vulnerability to Primary Progressive Multiple Sclerosis

Supplementary Figures

Supplementary Methods

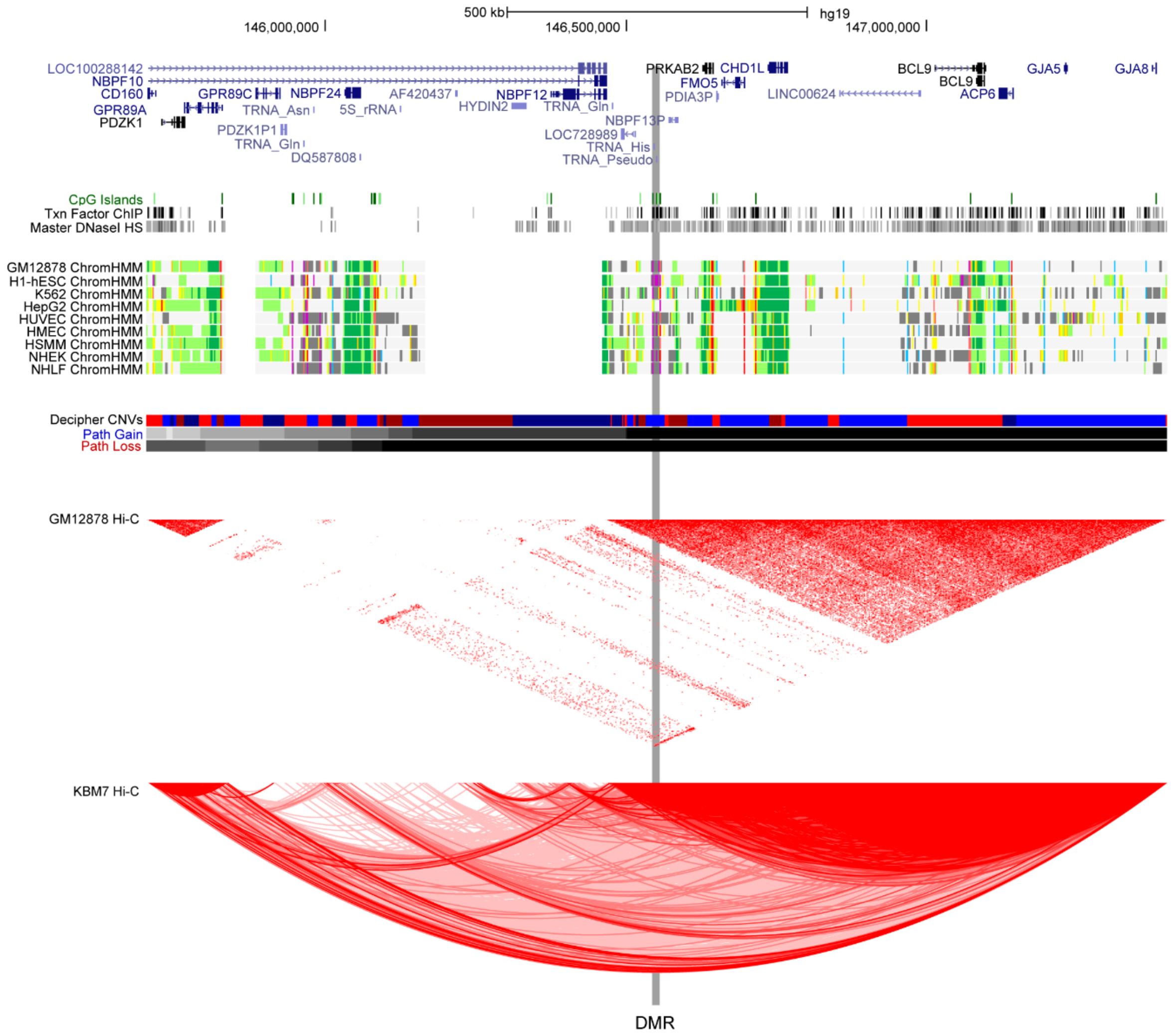

Supplementary Fig. 1. Chromatin chromosome interaction (Hi-C) annotation of the locus from Roadmap. The Hi-C annotation showing chromatin folding data from in-situ experiments on several cell lines[1] displayed by UCSC genome browser. The data indicate how many interactions were detected between regions of the genome. Two cell lines were chosen as examples: the color shade in the triangle mode shows the proximity score for two genomic regions, intersection was further depicted using arcs drawn between the centers of interacting regions. A high score between two regions suggests that they are probably in close proximity in 3D space within the nucleus of a cell.

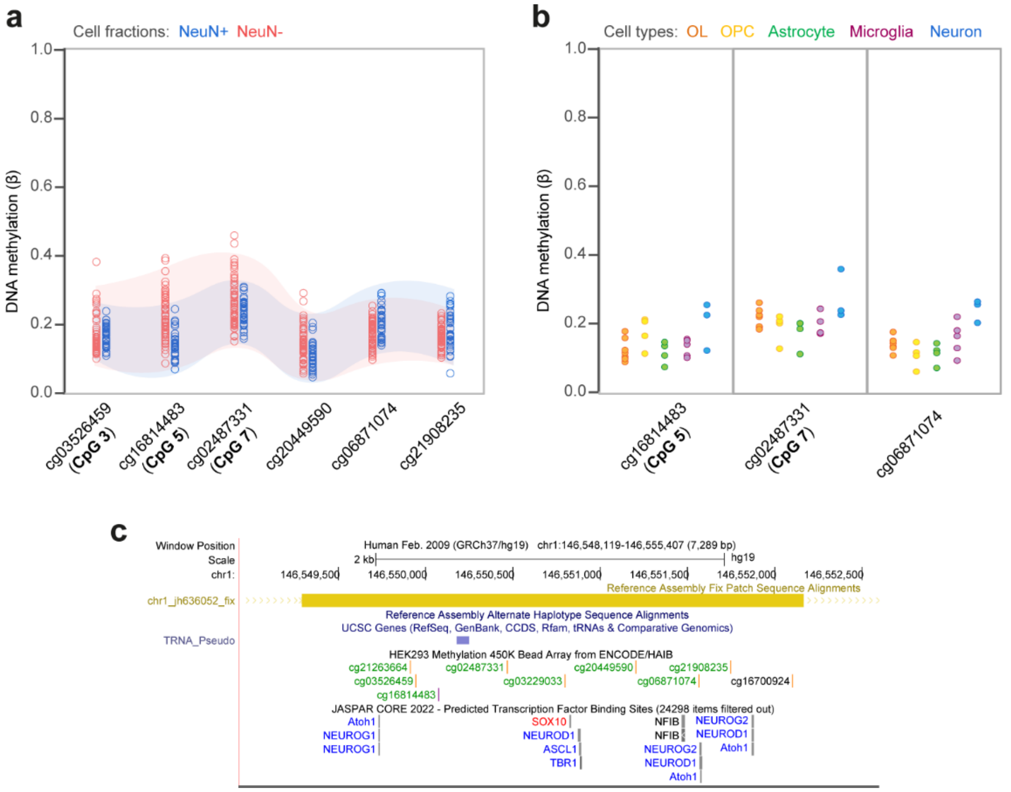

Supplementary Fig. 2. Methylation levels across brain cells and transcription factor binding sites in the 1q21.1 region. a. Methylation levels across CpGs in 1q21.1 region measured by Illumina arrays in l**a.** Sorted NeuN+ (neuronal, n = 34) and NeuN- (non-neuronal/glial, n = 56) nuclei from *postmortem* brain tissue (data from Kular *et al.*[2,3]) and **b.** Sorted and/or enriched brain cell types. Neurons (n = 3) comprise *ex vivo* sorted nuclei, oligodendrocytes (OL, n = 7) and microglia (n = 5) contain both *in vitro* expanded and *ex vivo* sorted samples, while oligodendrocyte progenitor cells (OPC, n = 4) and astrocytes (n = 4) are exclusively derived from *in vitro* primary cultures (genomic DNA purchased from Celprogen). **c.** JASPAR sequence analysis revealed binding sites for several transcription factors (TF) important for neuronal (blue), oligodendrocytic (red) and astrocytic (black) specification. The green colored CpGs indicate the hypermethylated CpGs found in PPMS, relative to RRMS and SPMS patients.

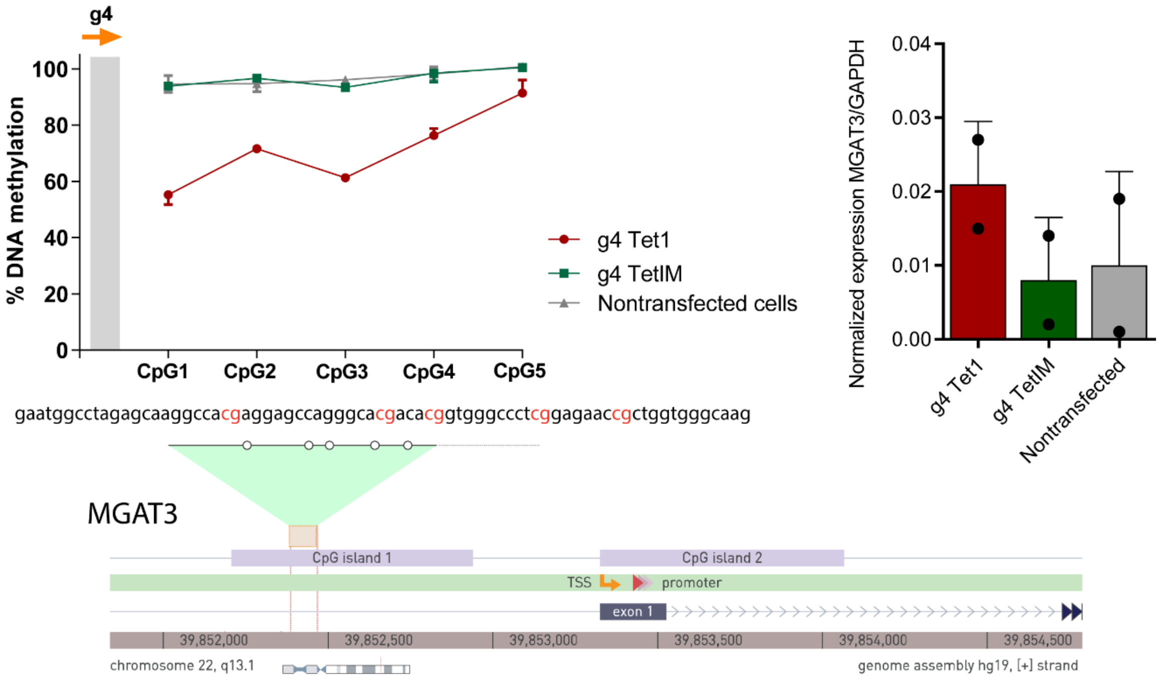

Supplementary Fig. 3. Validation of the editing efficiency of dCas9-TET1 constructs by targeting 5 CpGs in the *MGAT3* gene promoter. The methylation decreased 10-50% in different CpGs, resulting in no significant changes in *MGAT3* gene expression (Kruskal-Wallis test, P > 0.05 ±SD), as previously described[4].

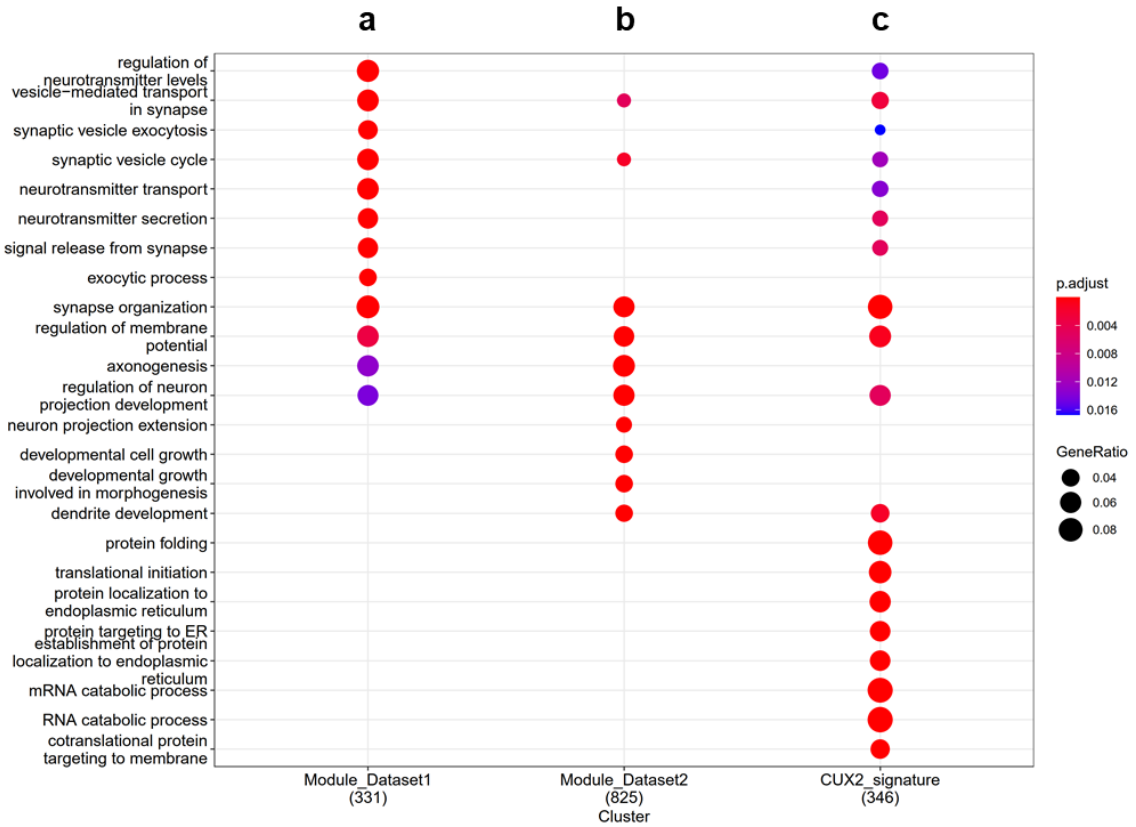

Supplementary Fig. 4. Enrichment dot plot showing Gene Ontology (GO): Biological processes for a. *CHD1L* module from our study. **b.** Validated module from snRNA-seq data (PRJNA544731) **c.** gene set for CUX2+ neuronal gene signature. The size of the dot corresponds to the gene ratio overlapping with the pathway and the color of the dot represents significance of the FDR p-value of the enrichment.

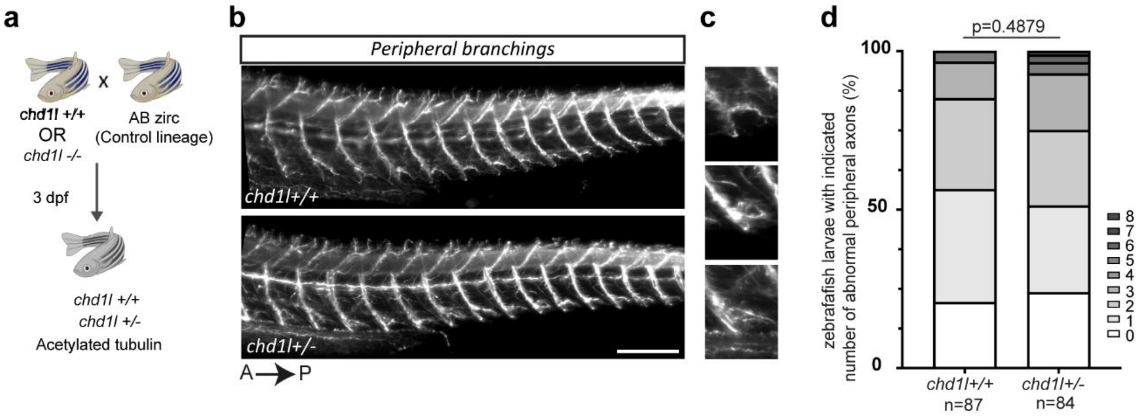

Supplementary Fig. 5. Effect of *chd1l* loss on peripheral axonal abnormalities in 3 dpf zebrafish larvae. **a.** Schematic of the experimental design. **b.** Lateral view of control *chd1l*+/+ and *chd1l*+/- larvae stained with acetylated tubulin at 3 dpf. Scale bar: 100 µm. **c.** Examples of axonal projections: normal branching (top), ectopic branching (middle) and reduced/absent branching (bottom) used for qualitative quantification of peripheral axons. **d.** Stacked barplot of the percentage of larvae with axonal projection defects in peripheral neurons. Number of abnormal projections were counted across eight metamers in control and *chd1l*+/- larvae. Data are expressed as percentage of larvae for N = 3 replicates for each genotype. Fischer's exact test is performed for p-value. A, antero; P, posterior, dpf: days post-fertilization. Source data are provided as a Source Data file.

>1q21.1 DMR_1st (CpG 1&2 in 450K) [285 bps]
GGGATGTTAAGGTTTGAGTTTTTTATGATATTCGGGGTGTTTCGATTTTATTTTGGGAGTTTTT
TCGTTTTTGTTTGTTTTTGGTTATTTAGTGCGCGGTTTTGGTTCGGTGATTCGTTATTGTTTTT
TTATGCGTTGTCGTTTTATAAAGGCGTATTTTTAGGTCGGTGGTTAGGTTTTGTTCGGACGTT
GTAATTGGTTTCGGGATTTGTGTTTTTGGTGAGTTTAGCGGAGTGTTGGGTGCGTCGTTTGC
GTGTTTTTTTTTTGGAGAAAGGAGGGAGGGAA

>1q21.1 DMR_2st (CpG 3 in 450K) [265 bps]
GTTGTAATTGGTTTCGGGATTTGTGTTTTTGGTGAGTTTAGCGGAGTGTTGGGTGCGTCGTT
TGCGTGTTTTTTTTTTGGAGAAAGGAGGGAGGGAACGGTTTTGTGAGACGATTTTAGGAGCG
ATTAGCGATTTTTATAAGTTTTAAGTTTTTTTAGCGTATAGGGAAATTGTTATTTATAGGGAAA
TTGTCGTTTTAGTGGTAAGAAGGTTGAAAAAGTTTTTTTTGTGTTTTTTTGGTGGTTTGGTGGT
TAGAATTTAGCGT

>1q21.1 DMR_3st (CpG 4 in 450K) [131 bps]
GGTAATTTTTGGTGGGTTATTTTTTATGATATAGCGTTTTTATTTATTTTCGTGTTTGTTATTCG
TATAAGCGGTTTTAGAGAGCGATTGAGCGTTTCGTTTAGGTGTATATCGTTGTGTAGAGATGT
TAG

>MGAT3 [118 bps]
GTTTTTGAGTTTTGAGAGGAATGGTTTAGAGTAAGGTTACGAGGAGTTAGGGTACGATACGG
TGGGTTTTCGGAGAATCGTTGGTGGGTAAGTGGTAGGAGAGTAGGTTTAAGAGGGT

Supplementary Fig. 6. Bisulfite amplicons for the pyrosequencing assays. CpG sites are highlighted in yellow. Sequencing primers are underlined.

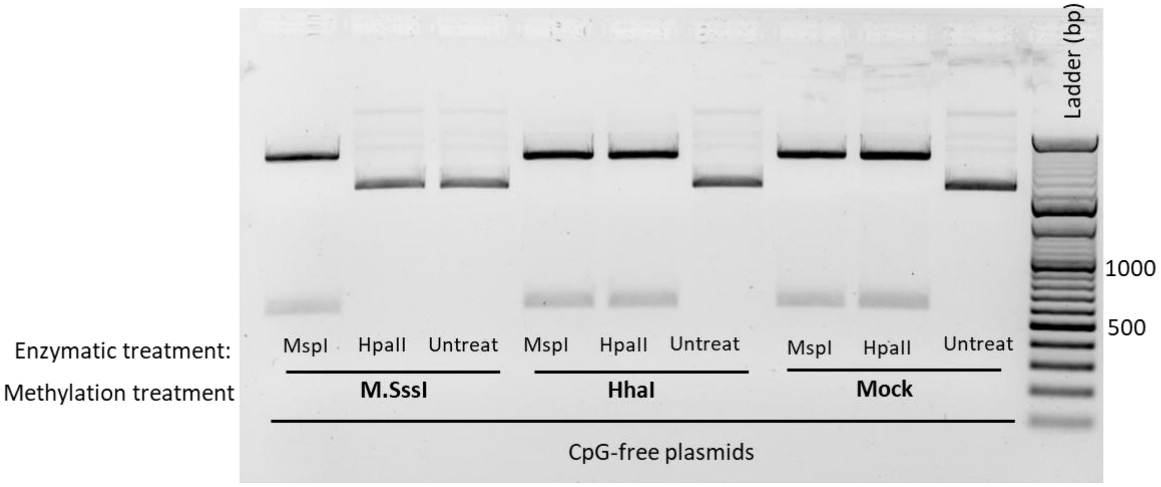

Supplementary Fig. 7. Methylation of 1q21.1 DMR vector used for in-vitro DNA methylation assay. Representative gel of completely methylated (57 CpGs) by *M.SssI* or partially methylated (7 CpGs residing in the GCGC sequence) by *HhaI* methyltransferases 1q21.1 DMR containing vector and empty constructs, treated with the methyl-insensitive MspI or the methyl-sensitive HpaII. The mock control was treated equally but in absence of any methyltransferases and correspond to unmethylated inserts.

P3-dCas9-Tet1-GFP-Puro (Addgene #190728)

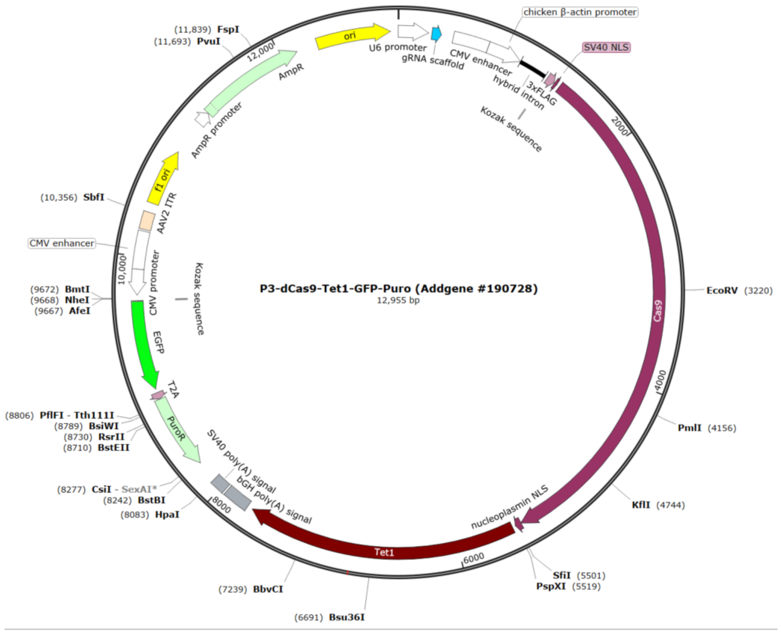

gagggcctatttcccatgattccttcatatttgcatatacgatacaaggctgttagagagataattggaattaatttgactgtaaacacaaag
atattagtacaaaatacgtgacgtagaaagtaataatttcttgggtagtttgcagttttaaaattatgttttaaaatggactatcatatgcttacc
gtaacttgaaagtatttcgatttcttggctttatatatcttgtggaaaggacgaaacaccgggtcttcgagaagacctgttttagagctagaaa
tagcaagttaaaataaggctagtccgttatcaacttgaaaaagtggcaccgagtcggtgctttttttgttttagagctagaaatagcaagttaa
aataaggctagtccgtttttagcgcgtgcgccaattctgcagacaaatggctctagaggtacccgttacataacttacggtaaatggcccg
cctggctgaccgcccaacgacccccgcccattgacgtcaatagtaacgccaatagggactttccattgacgtcaatgggtggagtattta
cggtaaactgcccacttggcagtacatcaagtgtatcatatgccaagtacgccccctattgacgtcaatgacggtaaatggcccgcctgg
cattgtgcccagtacatgaccttatgggactttcctacttggcagtacatctacgtattagtcatcgctattaccatggtcgaggtgagcccca
cgttctgcttcactctccccatctcccccccctccccacccccaattttgtatttatttattttttaattattttgtgcagcgatgggggcggggggg
gggggggggcgcgcgccaggcggggcggggcggggcgaggggcggggcggggcgaggcggagaggtgcggcggcagcca
atcagagcggcgcgctccgaaagtttccttttatggcgaggcggcggcggcggcggccctataaaaagcgaagcgcgcggcgggcg
ggagtcgctgcgcgctgccttcgccccgtgccccgctccgccgccgcctcgcgccgcccgccccggctctgactgaccgcgttactccc
acaggtgagcgggcgggacggcccttctcctccgggctgtaattagctgagcaagaggtaagggtttaagggatggttggttggtgggg
tattaatgtttaattacctggagcacctgcctgaaatcactttttttcaggttggaccggtgccaccatggactataaggaccacgacggag
actacaaggatcatgatattgattacaaagacgatgacgataagatggccccaaagaagaagcggaaggtcggtatccacggagtc
ccagcagccgacaagaagtacagcatcggcctggccatcggcaccaactctgtgggctgggccgtgatcaccgacgagtacaaggt
gcccagcaagaaattcaaggtgctgggcaacaccgaccggcacagcatcaagaagaacctgatcggagccctgctgttcgacagc
ggcgaaacagccgaggccacccggctgaagagaaccgccagaagaagatacaccagacggaagaaccggatctgctatctgca
agagatcttcagcaacgagatggccaaggtggacgacagcttcttccacagactggaagagtccttcctggtggaagaggataagaa
gcacgagcggcaccccatcttcggcaacatcgtggacgaggtggcctaccacgagaagtaccccaccatctaccacctgagaaaga
aactggtggacagcaccgacaaggccgacctgcggctgatctatctggccctggcccacatgatcaagttccggggccacttcctgatc
gagggcgacctgaaccccgacaacagcgacgtggacaagctgttcatccagctggtgcagacctacaaccagctgttcgaggaaaa
ccccatcaacgccagcggcgtggacgccaaggccatcctgtctgccagactgagcaagagcagacggctggaaaatctgatcgccc
agctgcccggcgagaagaagaatggcctgttcggcaacctgattgccctgagcctgggcctgacccccaacttcaagagcaacttcga
cctggccgaggatgccaaactgcagctgagcaaggacacctacgacgacgacctggacaacctgctggcccagatcggcgaccag
tacgccgacctgtttctggccgccaagaacctgtccgacgccatcctgctgagcgacatcctgagagtgaacaccgagatcaccaagg
ccccccctgagcgcctctatgatcaagagatacgacgagcaccaccaggacctgaccctgctgaaagctctcgtgcggcagcagctgc
ctgagaagtacaaagagattttcttcgaccagagcaagaacggctacgccggctacattgacggcggagccagccaggaagagttct
acaagttcatcaagcccatcctggaaaagatggacggcaccgaggaactgctcgtgaagctgaacagagaggacctgctgcggaa
gcagcggaccttcgacaacggcagcatcccccaccagatccacctgggagagctgcacgccattctgcggcggcaggaagatttta
cccattcctgaaggacaaccgggaaaagatcgagaagatcctgaccttccgcatcccctactacgtgggccctctggccaggggaaa
cagcagattcgcctggatgaccagaaagagcgaggaaaccatcacccccctggaacttcgaggaagtggtggacaagggcgcttccg

cccagagcttcatcgagcggatgaccaacttcgataagaacctgcccaacgagaaggtgctgcccaagcacagcctgctgtacgagt
acttcaccgtgtataacgagctgaccaaagtgaaatacgtgaccgagggaatgagaaagcccgccttcctgagcggcgagcagaaa
aaggccatcgtggacctgctgttcaagaccaaccggaaagtgaccgtgaagcagctgaaagaggactacttcaagaaaatcgagtg
cttcgactccgtggaaatctccggcgtggaagatcggttcaacgcctccctgggcacataccacgatctgctgaaaattatcaaggaca
aggacttcctggacaatgaggaaaacgaggacattctggaagatatcgtgctgaccctgacactgtttgaggacagagagatgatcga
ggaacggctgaaaacctatgcccacctgttcgacgacaaagtgatgaagcagctgaagcggcggagatacaccggctggggcagg
ctgagccggaagctgatcaacggcatccgggacaagcagtccggcaagacaatcctggatttcctgaagtccgacggcttcgccaac
agaaacttcatgcagctgatccacgacgacagcctgacctttaaagaggacatccagaaagcccaggtgtccggccagggcgatag
cctgcacgagcacattgccaatctggccggcagccccgccattaagaagggcatcctgcagacagtgaaggtggtggacgagctcgt
gaaagtgatgggccggcacaagcccgagaacatcgtgatcgaaatggccagagagaaccagaccacccagaagggacagaag
aacagccgcgagagaatgaagcggatcgaagagggcatcaaagagctgggcagccagatcctgaaagaacaccccgtggaaaa
cacccagctgcagaacgagaagctgtacctgtactacctgcagaatgggcgggatatgtacgtggaccaggaactggacatcaaccg
gctgtccgactacgatgtggacgctatcgtgcctcagagctttctgaaggacgactccatcgacaacaaggtgctgaccagaagcgac
aagaaccggggcaagagcgacaacgtgccctccgaagaggtcgtgaagaagatgaagaactactggcggcagctgctgaacgcc
aagctgattacccagagaaagttcgacaatctgaccaaggccgagagaggcggcctgagcgaactggataaggccggcttcatcaa
gagacagctggtggaaacccggcagatcacaaagcacgtggcacagatcctggactcccggatgaacactaagtacgacgagaat
gacaagctgatccgggaagtgaaagtgatcaccctgaagtccaagctggtgtccgatttccggaaggatttccagttttacaaagtgcgc
gagatcaacaactaccaccacgcccacgacgcctacctgaacgccgtcgtgggaaccgccctgatcaaaaagtaccctaagctgga
aagcgagttcgtgtacggcgactacaaggtgtacgacgtgcggaagatgatcgccaagagcgagcaggaaatcggcaaggctacc
gccaagtacttcttctacagcaacatcatgaactttttcaagaccgagattaccctggccaacggcgagatccggaagcggcctctgatc
gagacaaacggcgaaaccggggagatcgtgtgggataagggccgggattttgccaccgtgcggaaagtgctgagcatgccccaagt
gaatatcgtgaaaaagaccgaggtgcagacaggcggcttcagcaaagagtctatcctgcccaagaggaacagcgataagctgatcg
ccagaaagaaggactgggaccctaagaagtacggcggcttcgacagccccaccgtggcctattctgtgctggtggtggccaaagtgg
aaaagggcaagtccaagaaactgaagagtgtgaaagagctgctggggatcaccatcatggaaagaagcagcttcgagaagaatcc
catcgactttctggaagccaagggctacaaagaagtgaaaaaggacctgatcatcaagctgcctaagtactccctgttcgagctggaa
aacggccggaagagaatgctggcctctgccggcgaactgcagaagggaaacgaactggccctgccctccaaatatgtgaacttcctg
tacctggccagccactatgagaagctgaagggctcccccgaggataatgagcagaaacagctgtttgtggaacagcacaagcactac
ctggacgagatcatcgagcagatcagcgagttctccaagagagtgatcctggccgacgctaatctggacaaagtgctgtccgcctaca
acaagcaccgggataagcccatcagagagcaggccgagaatatcatccacctgtttaccctgaccaatctgggagcccctgccgcctt
caagtactttgacaccaccatcgaccggaagaggtacaccagcaccaaagaggtgctggacgccaccctgatccaccagagcatca
ccggcctgtacgagacacggatcgacctgtctcagctgggaggcgacaaaaggccggcggccacgaaaaaggccggacaggcc
aaaaagaaaaagctcgagggcggaggcgggagcggatccctgcccacctgcagctgtcttgatcgagttatacaaaaagacaaag
gcccatattatacacaccttggggcaggaccaagtgttgctgctgtcagggaaatcatggagaataggtatggtcaaaaaggaaacgc
aataaggatagaaatagtagtgtacaccggtaaagaagggaaaagctctcatgggtgtccaattgctaagtgggttttaagaagaagc
agtgatgaagaaaaagttctttgtttggtccggcagcgtacaggccaccactgtccaactgctgtgatggtggtgctcatcatggtgtgga
tggcatccctcttccaatggccgaccggctatacacagagctcacagagaatctaaagtcatacaatgggcaccctaccgacagaag
atgcaccctcaatgaaaatcgtacctgtacatgtcaaggaattgatccagagacttgtggagcttcattctcttttggctgttcatggagtatgt
actttaatggctgtaagtttggtagaagcccaagccccagaagatttagaattgatccaagctctcccttacatgaaaaaaaccttgaaga
taacttacagagtttggctacacgattagctccaatttataagcagtatgctccagtagcttaccaaaatcaggtggaatatgaaaatgttg
cccgagaatgtcggcttggcagcaaggaaggtcgacccttctctggggtcactgcttgcctggacttctgtgctcatccccacagggacat
tcacaacatgaataatggaagcactgtggtttgtaccttaactcgagaagataaccgctctttgggtgttattcctcaagatgagcagctcc
atgtgctacctctttataagctttcagacacagatgagtttggctccaaggaaggaatggaagccaagatcaaatctggggccatcgagg
tcctggcaccccgccgcaaaaaaagaacgtgtttcactcagcctgttccccgttctggaaagaagagggctgcgatgatgacagaggtt
cttgcacataagataagggcagtggaaaagaaacctattccccgaatcaagcggaagaataactcaacaacaacaaacaacagta
agccttcgtcactgccaaccttagggagtaacactgagaccgtgcaacctgaagtaaaaagtgaaaccgaaccccattttatcttaaaa
agttcagacaacactaaaacttattcgctgatgccatccgctcctcacccagtgaaagaggcatctccaggcttctcctggtcccccaag
actgcttcagccacaccagctccactgaagaatgacgcaacagcctcatgcgggttttcagaaagaagcagcactccccactgtacga
tgccttcgggtagactcagtggtgccaatgctgcagctgctgatggccctggcatttcacagcttggcgaagtggctcctctccccaccctg
tctgctcctgtgatggagcccctcattaattctgagccttccactggtgtgactgagccgctaacgcctcatcagccaaaccaccagccctc
cttcctcacctctcctcaagaccttgcctcttctccaatggaagaagatgagcagcattctgaagcagatgagcctccatcagacgaacc
cctatctgatgacccccctgtcacctgctgaggagaaattgccccacattgatgagtattggtcagacagtgagcacatctttttggatgcaa
atattggtggggtggccatcgcacctgctcacggctcggttttgattgagtgtgcccggcgagagctgcacgctaccactcctgttgagca
ccccaaccgtaatcatccaacccgcctctcccttgtcttttaccagcacaaaaacctaaataagccccaacatggttttgaactaaacaa
gattaagtttgaggctaaagaagctaagaataagaaaatgaaggcctcagagcaaaaagaccaggcagctaatgaaggtccagaa
cagtcctctgaagtaaatgaattgaaccaaattccttctcataaagcattaacattaacccatgacaatgttgtcaccgtgtccccttatgctc
tcacacacgttgcggggccctataaccattgggtctgagaattcaattctaactagagctcgctgatcagcctcgactgtgccttctagttgc

cagccatctgttgtttgcccctcccccgtgccttccttgaccctggaaggtgccactcccactgtcctttcctaataaaatgaggaaattgcat
cgcattgtctgagtaggtgtcattctattctggggggtggggtggggcaggacagcaagggggaggattgggaagagaatagcaggc
atgctggggagcggccgccgcgttaagatacattgatgagtttggacaaaccacaactagaatgcagtgaaaaaaatgctttatttgtga
aatttgtgatgctattgctttatttgtaaccattataagctgcaataaacaagttaacaacaacaattgcattcattttatgtttcaggttcagggg
gaggtgtgggaggttttttaaagcaagtaaaacctctacaaatgtggtatggctgattatgatcagttatctagatccggtggatcccgggc
ccgcggtaccgtcgactgcagaattcgaagctttcaggcaccgggcttgcgggtcatgcaccaggtgcgcggtccttcgggcacctcga
cgtcggcggtgacggtgaagccgagccgctcgtagaaggggaggttgtggggcgcggaggtctccaggaaggcgggcaccccgg
cgcgctcggccgcctccactccggggagcacgacggcgctgcccagacccttgccctggtggtcgggcgagacgccgacggtggcc
aggaaccacgcgggctccttgggccggtgcggcgccaggaggccttccatctgttgctgcgcggccagccgggaaccgctcaactcg
gccatgcgcgggccgatctcggcgaacaccgcccccgcttcgacgctctccggcgtggtccagaccgccaccgcggcgccgtcgtcc
gcgacccacaccttgccgatgtcgagcccgacgcgcgtgaggaagagttcttgcagctcggtgacccgctcgatgtggcggtccggat
cgacggtgtggcgcgtggcggggtagtcggcgaacgcggcggcgagggtgcgtacggccctggggacgtcgtcgcgggtggcgag
gcgcaccgtgggcttgtactcggtcattgggccaggattctcctcgacgtcaccgcatgttagcagacttcctctgccctcagaagcttgag
ctcgagatctgagtccggacttgtacagctcgtccatgccgagagtgatcccggcggcggtcacgaactccagcaggaccatgtgatc
gcgcttctcgttggggtctttgctcagggcggactgggtgctcaggtagtggttgtcgggcagcagcacggggccgtcgccgatgggggt
gttctgctggtagtggtcggcgagctgcacgctgccgtcctcgatgttgtggcggatcttgaagttcaccttgatgccgttcttctgcttgtcgg
ccatgatatagacgttgtggctgttgtagttgtactccagcttgtgccccaggatgttgccgtcctccttgaagtcgatgcccttcagctcgatg
cggttcaccagggtgtcgccctcgaacttcacctcggcgcgggtcttgtagttgccgtcgtccttgaagaagatggtgcgctcctggacgt
agccttcgggcatggcggacttgaagaagtcgtgctgcttcatgtggtcggggtagcggctgaagcactgcacgccgtaggtcagggtg
gtcacgagggtgggccagggcacgggcagcttgccggtggtgcagatgaacttcagggtcagcttgccgtaggtggcatcgccctcgc
cctcgccggacacgctgaacttgtggccgtttacgtcgccgtccagctcgaccaggatgggcaccaccccggtgaacagctcctcgcc
cttgctcaccatggtggcgaccggtagcgctagcggatctgacggttcactaaaccagctctgcttatatagacctcccaccgtacacgc
ctaccgcccatttgcgtcaatggggcggagttgttacgacattttggaaagtcccgttgattttggtgccaaaacaaactcccattgacgtca
atggggtggagacttggaaatccccgtgagtcaaaccgctatccacgcccattgatgtactgccaaaaccgcatcaccatggtaatagc
gatgactaatacgtagatgtactgccaagtaggaaagtcccataaggtcatgtactgggcacaatgccaggcgggccatttaccgtcatt
gacgtcaatagggggcgtacttggcatatgatacacttgatgtactgccaagtgggcagtttaccgtaaatactccacccattgacgtcaa
tggaaagtccctattggcgttactatgggaacatacgtcattattgacgtcaatgggcgggggtcgttgggcggtcagccaggcgggcca
tttaccgtaagttatgtaacgcggaacgcggccgcaggaaccccctagtgatggagttggccactccctctctgcgcgctcgctcgctcact
gaggccgggcgaccaaaggtcgcccgacgcccgggctttgcccgggcggcctcagtgagcgagcgagcgcgcagctgcctgcag
gggcgcctgatgcggtattttctccttacgcatctgtgcggtatttcacaccgcatacgtcaaagcaaccatagtacgcgccctgtagcggc
gcattaagcgcggcgggtgtggtggttacgcgcagcgtgaccgctacacttgccagcgccttagcgcccgctcctttcgctttcttcccttcc
tttctcgccacgttcgccggctttccccgtcaagctctaaatcgggggctccctttagggttccgatttagtgctttacggcacctcgacccca
aaaaacttgatttgggtgatggttcacgtagtgggccatcgccctgatagacggtttttcgccctttgacgttggagtccacgttctttaatagt
ggactcttgttccaaactggaacaacactcaactctatctcgggctattcttttgatttataagggattttgccgatttcggtctattggttaaaaa
atgagctgatttaacaaaaatttaacgcgaattttaacaaaatattaacgtttacaattttatggtgcactctcagtacaatctgctctgatgcc
gcatagttaagccagccccgacacccgccaacacccgctgacgcgccctgacgggcttgtctgctcccggcatccgcttacagacaa
gctgtgaccgtctccgggagctgcatgtgtcagaggttttcaccgtcatcaccgaaacgcgcgagacgaaagggcctcgtgatacgcct
atttttataggttaatgtcatgataataatggtttcttagacgtcaggtggcacttttcggggaaatgtgcgcggaacccctatttgtttatttttcta
aatacattcaaatatgtatccgctcatgagacaataaccctgataaatgcttcaataatattgaaaaaggaagagtatgagtattcaacatt
tccgtgtcgcccttattcccttttttgcggcattttgccttcctgtttttgctcacccagaaacgctggtgaaagtaaaagatgctgaagatcagtt
gggtgcacgagtgggttacatcgaactggatctcaacagcggtaagatccttgagagttttcgccccgaagaacgttttccaatgatgag
cacttttaaagttctgctatgtggcgcggtattatcccgtattgacgccgggcaagagcaactcggtcgccgcatacactattctcagaatg
acttggttgagtactcaccagtcacagaaaagcatcttacggatggcatgacagtaagagaattatgcagtgctgccataaccatgagtg
ataacactgcggccaacttacttctgacaacgatcggaggaccgaaggagctaaccgcttttttgcacaacatgggggatcatgtaactc
gccttgatcgttgggaaccggagctgaatgaagccataccaaacgacgagcgtgacaccacgatgcctgtagcaatggcaacaacgt
tgcgcaaactattaactggcgaactacttactctagcttcccggcaacaattaatagactggatggaggcggataaagttgcaggacca
cttctgcgctcggcccttccggctggctggtttattgctgataaatctggagccggtgagcgtggaagccgcggtatcattgcagcactggg
gccagatggtaagccctcccgtatcgtagttatctacacgacggggagtcaggcaactatggatgaacgaaatagacagatcgctgag
ataggtgcctcactgattaagcattggtaactgtcagaccaagtttactcatatatactttagattgatttaaaacttcatttttaatttaaaagga
tctaggtgaagatcctttttgataatctcatgaccaaaatcccttaacgtgagttttcgttccactgagcgtcagaccccgtagaaaagatca
aaggatcttcttgagatcctttttttctgcgcgtaatctgctgcttgcaaacaaaaaaaccaccgctaccagcggtggtttgtttgccggatca
agagctaccaactctttttccgaaggtaactggcttcagcagagcgcagataccaaatactgttcttctagtgtagccgtagttaggccacc
acttcaagaactctgtagcaccgcctacatacctcgctctgctaatcctgttaccagtggctgctgccagtggcgataagtcgtgtcttaccg
ggttggactcaagacgatagttaccggataaggcgcagcggtcgggctgaacggggggttcgtgcacacagcccagcttggagcga
acgacctacaccgaactgagatacctacagcgtgagctatgagaaagcgccacgcttcccgaagggagaaaggcggacaggtatc
cggtaagcggcagggtcggaacaggagagcgcacgagggagcttccagggggaaacgcctggtatctttatagtcctgtcgggtttcg

ccacctctgacttgagcgtcgatttttgtgatgctcgtcaggggggcggagcctatggaaaaacgccagcaacgcggcctttttacggttc
ctggccttttgctggccttttgctcacatgt

P3-Lenti-dCas9-Tet1-GFP (Addgene #190729)

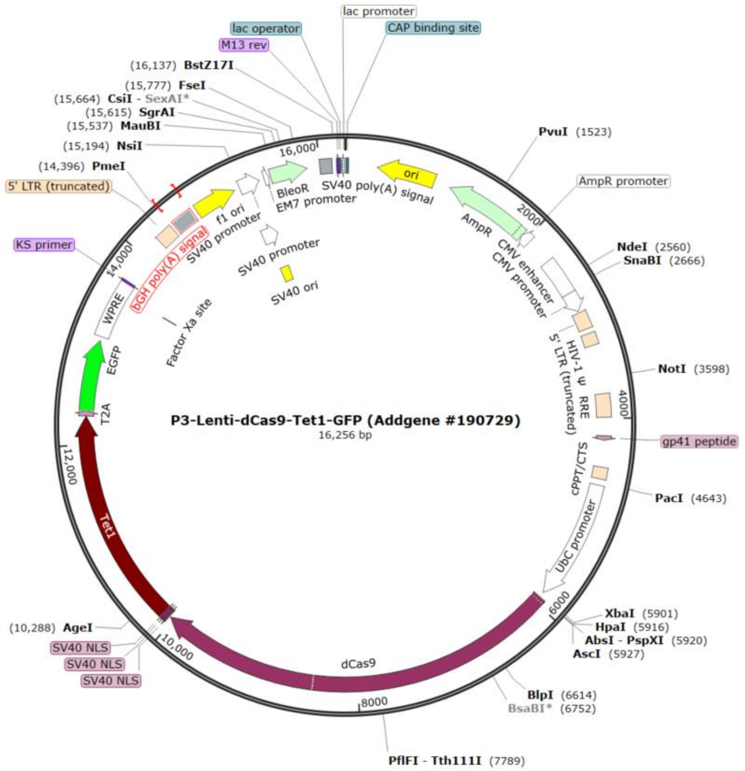

tggggtgcctaatgagtgagctaactcacattaattgcgttgcgctcactgcccgctttccagtcgggaaacctgtcgtgccagctgcatta
atgaatcggccaacgcgcggggagaggcggtttgcgtattgggcgctcttccgcttcctcgctcactgactcgctgcgctcggtcgttcgg
ctgcggcgagcggtatcagctcactcaaaggcggtaatacggttatccacagaatcaggggataacgcaggaaagaacatgtgagc
aaaaggccagcaaaaggccaggaaccgtaaaaaggccgcgttgctggcgtttttccataggctccgcccccctgacgagcatcaca
aaaatcgacgctcaagtcagaggtggcgaaacccgacaggactataaagataccaggcgtttccccctggaagctccctcgtgcgct
ctcctgttccgaccctgccgcttaccggatacctgtccgcctttctcccttcgggaagcgtggcgctttctcatagctcacgctgtaggtatctc
agttcggtgtaggtcgttcgctccaagctgggctgtgtgcacgaaccccccgttcagcccgaccgctgcgccttatccggtaactatcgtct
tgagtccaacccggtaagacacgacttatcgccactggcagcagccactggtaacaggattagcagagcgaggtatgtaggcggtgc
tacagagttcttgaagtggtggcctaactacggctacactagaagaacagtatttggtatctgcgctctgctgaagccagttaccttcggaa
aaagagttggtagctcttgatccggcaaacaaaccaccgctggtagcggtggtttttttgtttgcaagcagcagattacgcgcagaaaaa
aaggatctcaagaagatcctttgatcttttctacggggtctgacgctcagtggaacgaaaactcacgttaagggattttggtcatgagattat
caaaaaggatcttcacctagatccttttaaattaaaaatgaagttttaaatcaatctaaagtatatatgagtaaacttggtctgacagttacca
atgcttaatcagtgaggcacctatctcagcgatctgtctatttcgttcatccatagttgcctgactccccgtcgtgtagataactacgatacgg
gagggcttaccatctggccccagtgctgcaatgataccgcgagacccacgctcaccggctccagatttatcagcaataaaccagccag
ccggaagggccgagcgcagaagtggtcctgcaactttatccgcctccatccagtctattaattgttgccgggaagctagagtaagtagttc
gccagttaatagtttgcgcaacgttgttgccattgctacaggcatcgtggtgtcacgctcgtcgtttggtatggcttcattcagctccggttccc
aacgatcaaggcgagttacatgatcccccatgttgtgcaaaaaagcggttagctccttcggtcctccgatcgttgtcagaagtaagttggc
cgcagtgttatcactcatggttatggcagcactgcataattctcttactgtcatgccatccgtaagatgcttttctgtgactggtgagtactcaac
caagtcattctgagaatagtgtatgcggcgaccgagttgctcttgcccggcgtcaatacgggataataccgcgccacatagcagaacttt
aaaagtgctcatcattggaaaacgttcttcggggcgaaaactctcaaggatcttaccgctgttgagatccagttcgatgtaacccactcgt
gcacccaactgatcttcagcatcttttactttcaccagcgtttctgggtgagcaaaaacaggaaggcaaaatgccgcaaaaaagggaat
aagggcgacacggaaatgttgaatactcatactcttcctttttcaatattattgaagcatttatcagggttattgtctcatgagcggatacatatt
tgaatgtatttagaaaaataaacaaataggggttccgcgcacatttccccgaaaagtgccacctgacgtcgacggatcgggagatctcc
cgatcccctatggtgcactctcagtacaatctgctctgatgccgcatagttaagccagtatctgctccctgcttgtgtgttggaggtcgctgag
tagtgcgcgagcaaaatttaagctacaacaaggcaaggcttgaccgacaattgcatgaagaatctgcttagggttaggcgttttgcgctg
cttcgcgatgtacgggccagatatacgcgttgacattgattattgactagttattaatagtaatcaattacggggtcattagttcatagcccat
atatggagttccgcgttacataacttacggtaaatggcccgcctggctgaccgcccaacgacccccgcccattgacgtcaataatgacgt
atgttcccatagtaacgccaatagggactttccattgacgtcaatgggtggagtatttacggtaaactgcccacttggcagtacatcaagtg
tatcatatgccaagtacgccccctattgacgtcaatgacggtaaatggcccgcctggcattatgcccagtacatgaccttatgggactttcc

tacttggcagtacatctacgtattagtcatcgctattaccatggtgatgcggttttggcagtacatcaatgggcgtggatagcggtttgactca
cggggatttccaagtctccaccccattgacgtcaatgggagtttgttttggcaccaaaatcaacgggactttccaaaatgtcgtaacaactc
cgccccattgacgcaaatgggcggtaggcgtgtacggtgggaggtctatataagcagcgcgttttgcctgtactgggtctctctggttaga
ccagatctgagcctgggagctctctggctaactagggaacccactgcttaagcctcaataaagcttgccttgagtgcttcaagtagtgtgtg
cccgtctgttgtgtgactctggtaactagagatccctcagacccttttagtcagtgtggaaaatctctagcagtggcgcccgaacagggact
tgaaagcgaaagggaaaccagaggagctctctcgacgcaggactcggcttgctgaagcgcgcacggcaagaggcgaggggcgg
cgactggtgagtacgccaaaaattttgactagcggaggctagaaggagagagatgggtgcgagagcgtcagtattaagcgggggag
aattagatcgcgatgggaaaaaattcggttaaggccagggggaaagaaaaaatataaattaaaacatatagtatgggcaagcaggg
agctagaacgattcgcagttaatcctggcctgttagaaacatcagaaggctgtagacaaatactgggacagctacaaccatcccttcag
acaggatcagaagaacttagatcattatataatacagtagcaaccctctattgtgtgcatcaaaggatagagataaaagacaccaagg
aagctttagacaagatagaggaagagcaaaacaaaagtaagaccaccgcacagcaagcggccgctgatcttcagacctggagga
ggagatatgagggacaattggagaagtgaattatataaatataaagtagtaaaaattgaaccattaggagtagcacccaccaaggca
aagagaagagtggtgcagagagaaaaaagagcagtgggaataggagctttgttccttgggttcttgggagcagcaggaagcactatg
ggcgcagcgtcaatgacgctgacggtacaggccagacaattattgtctggtatagtgcagcagcagaacaatttgctgagggctattga
ggcgcaacagcatctgttgcaactcacagtctggggcatcaagcagctccaggcaagaatcctggctgtggaaagatacctaaagga
tcaacagctcctggggatttggggttgctctggaaaactcatttgcaccactgctgtgccttggaatgctagttggagtaataaatctctgga
acagatttggaatcacacgacctggatggagtgggacagagaaattaacaattacacaagcttaatacactccttaattgaagaatcgc
aaaaccagcaagaaaagaatgaacaagaattattggaattagataaatgggcaagtttgtggaattggtttaacataacaaattggctg
tggtatataaaattattcataatgatagtaggaggcttggtaggtttaagaatagtttttgctgtactttctatagtgaatagagttaggcaggg
atcccaaccccgaggggacccgacaggcccgaaggaatagaagaagaaggtggagagagagacagagacagatccattcgatt
agtgaacggatcggcactgcgtgcgccaattctgcagacaaatggcagtattcatccacaattttaaaagaaaaggggggattgggg
gtacagtgcaggggaaagaatagtagacataatagcaacagacatacaaactaaagaattacaaaaacaaattacaaaaattcaa
aattttcgggtttattacagggacagcagagatccagtttggttaattaacccgtgtcggctccagatctggcctccgcgccgggttttggcg
cctcccgcgggcgcccccctcctcacggcgagcgctgccacgtcagacgaagggcgcagcgagcgtcctgatccttccgcccggac
gctcaggacagcggcccgctgctcataagactcggccttagaaccccagtatcagcagaaggacattttaggacgggacttgggtgac
tctagggcactggttttctttccagagagcggaacaggcgaggaaaagtagtcccttctcggcgattctgcggagggatctccgtggggc
ggtgaacgccgatgattatataaggacgcgccgggtgtggcacagctagttccgtcgcagccgggatttgggtcgcggttcttgtttgtgg
atcgctgtgatcgtcacttggtgagtagcgggctgctgggctggccggggctttcgtggccgccgggccgctcggtgggacggaagcgt
gtggagagaccgccaagggctgtagtctgggtccgcgagcaaggttgccctgaactgggggttggggggagcgcagcaaaatggc
ggctgttcccgagtcttgaatggaagacgcttgtgaggcgggctgtgaggtcgttgaaacaaggtgggggggcatggtgggcggcaaga
acccaaggtcttgaggccttcgctaatgcgggaaagctcttattcgggtgagatgggctggggcaccatctggggaccctgacgtgaag
tttgtcactgactggagaactcggtttgtcgtctgttgcgggggcggcagttatggcggtgccgttgggcagtgcacccgtacctttgggag
cgcgcgccctcgtcgtgtcgtgacgtcacccgttctgttggcttataatgcagggtggggccacctgccggtaggtgtgcggtaggcttttct
ccgtcgcaggacgcagggttcgggcctagggtaggctctcctgaatcgacaggcgccggacctctggtgaggggagggataagtga
ggcgtcagtttctttggtcggttttatgtacctatcttcttaagtagctgaagctccggttttgaactatgcgctcggggttggcgagtgtgttttgt
gaagtttttaggcaccttttgaaatgtaatcatttgggtcaatatgtaattttcagtgttagactagtaaattgtccgctaaattctggccgtttttg
gctttttgttagacgaagcttgggctgcaggtcgactctagaggatccagttaacctcgagggcgcgcccatggacaagaagtattctat
cggactggccatcgggactaatagcgtcgggtgggccgtgatcactgacgagtacaaggtgccctctaagaagttcaaggtgctcggg
aacaccgaccggcattccatcaagaaaaatctgatcggagctctcctctttgattcaggggagaccgctgaagcaacccgcctcaagc
ggactgctagacggcggtacaccaggaggaagaaccggatttgttaccttcaagagatattctccaacgaaatggcaaaggtcgacg
acagcttcttccataggctggaagaatcattcctcgtggaagaggataagaagcatgaacggcatcccatcttcggtaatatcgtcgacg
aggtggcctatcacgagaaatacccaaccatctaccatcttcgcaaaaagctggtggactcaaccgacaaggcagacctccggcttat
ctacctggccctggcccacatgatcaagttcagaggccacttcctgatcgagggcgacctcaatcctgacaatagcgatgtggataaac
tgttcatccagctggtgcagacttacaaccagctctttgaagagaaccccatcaatgcaagcggagtcgatgccaaggccattctgtcag
cccggctgtcaaagagccgcagacttgagaatcttatcgctcagctgccgggtgaaaagaaaaatggactgttcgggaacctgattgct
ctttcacttgggctgactcccaatttcaagtctaatttcgacctggcagaggatgccaagctgcaactgtccaaggacacctatgatgacg
atctcgacaacctcctggcccagatcggtgaccaatacgccgaccttttccttgctgctaagaatctttctgacgccatcctgctgtctgaca
ttctccgcgtgaacactgaaatcaccaaggcccctctttcagcttcaatgattaagcggtatgatgagcaccaccaggacctgaccctgct
taaggcactcgtccggcagcagcttccggagaagtacaaggaaatcttctttgaccagtcaaagaatggatacgccggctacatcgac
ggaggtgcctcccaagaggaattttataagtttatcaaacctatccttgagaagatggacggcaccgaagagctcctcgtgaaactgaat
cgggaggatctgctgcggaagcagcgcactttcgacaatggagcattccccaccagatccatcttggggagcttcacgccatccttcg
gcgccaagaggacttctacccctttcttaaggacaacagggagaagattgagaaaattctcactttccgcatcccctactacgtgggacc
cctcgccagaggaaatagccggtttgcttggatgaccagaaagtcagaagaaactatcactccctggaacttcgaagaggtggtggac
aagggagccagcgctcagtcattcatcgaacggatgactaacttcgataagaacctccccaatgagaaggtcctgccgaaacattccc
tgctctacgagtactttaccgtgtacaacgagctgaccaaggtgaaatatgtcaccgaagggatgaggaagcccgcattcctgtcaggc
gaacaaaagaaggcaattgtggaccttctgttcaagaccaatagaaaggtgaccgtgaagcagctgaaggaggactatttcaagaaa

attgaatgcttcgactctgtggagattagcggggtcgaagatcggttcaacgcaagcctgggtacctaccatgatctgcttaagatcatca
aggacaaggattttctggacaatgaggagaacgaggacatccttgaggacattgtcctgactctcactctgttcgaggaccgggaaatg
atcgaggagaggcttaagacctacgcccatctgttcgacgataaagtgatgaagcaacttaaacggagaagatataccggatgggga
cgccttagccgcaaactcatcaacggaatccgggacaaacagagcggaaagaccattcttgatttccttaaaagcgacggattcgcta
atcgcaacttcatgcaacttatccatgatgattccctgacctttaaggaggacatccagaaggcccaagtgtctggacaaggtgactcact
gcacgagcatatcgcaaatctggctggttcacccgctattaagaagggtattctccagaccgtgaaagtcgtggacgagctggtcaagg
tgatgggtcgccataaaccagagaacattgtcatcgagatggccagggaaaaccagactacccagaagggacagaagaacagca
gggagcggatgaaaagaattgaggaagggattaaggagctcgggtcacagatccttaaagagcacccggtggaaaacacccagct
tcagaatgagaagctctatctgtactaccttcaaaatggacgcgatatgtatgtggaccaagagcttgatatcaacaggctctcagactac
gacgtggacgccatcgtccctcagagcttcctcaaagacgactcaattgacaataaggtgctgactcgctcagacaagaaccgggga
aagtcagataacgtgccctcagaggaagtcgtgaaaaagatgaagaactattggcgccagcttctgaacgcaaagctgatcactcag
cggaagttcgacaatctcactaaggctgagaggggcggactgagcgaactggacaaagcaggattcattaaacggcaacttgtgga
gactcggcagattactaaacatgtcgcccaaatccttgactcacgcatgaataccaagtacgacgaaaacgacaaacttatccgcgag
gtgaaggtgattaccctgaagtccaagctggtcagcgatttcagaaaggactttcaattctacaaagtgcgggagatcaataactatcatc
atgctcatgacgcatatctgaatgccgtggtgggaaccgccctgatcaagaagtacccaaagctggaaagcgagttcgtgtacggaga
ctacaaggtctacgacgtgcgcaagatgattgccaaatctgagcaggagatcggaaaggccaccgcaaagtacttcttctacagcaac
atcatgaatttcttcaagaccgaaatcacccttgcaaacggtgagatccggaagaggccgctcatcgagactaatggggagactggcg
aaatcgtgtgggacaagggcagagatttcgctaccgtgcgcaaagtgctttctatgcctcaagtgaacatcgtgaagaaaaccgaggtg
caaaccggaggcttttctaaggaatcaatcctccccaagcgcaactccgacaagctcattgcaaggaagaaggattgggaccctaag
aagtacggcggattcgattcaccaactgtggcttattctgtcctggtcgtggctaaggtggaaaaaggaaagtctaagaagctcaagag
cgtgaaggaactgctgggtatcaccattatggagcgcagctccttcgagaagaacccaattgactttctcgaagccaaaggttacaagg
aagtcaagaaggaccttatcatcaagctcccaaagtatagcctgttcgaactggagaatgggcggaagcggatgctcgcctccgctgg
cgaacttcagaagggtaatgagctggctctcccctccaagtacgtgaatttcctctaccttgcaagccattacgagaagctgaaggggag
ccccgaggacaacgagcaaaagcaactgtttgtggagcagcataagcattatctggacgagatcattgagcagatttccgagttttctaa
acgcgtcattctcgctgatgccaacctcgataaagtccttagcgcatacaataagcacagagacaaaccaattcgggagcaggctgag
aatatcatccacctgttcaccctcaccaatcttggtgcccctgccgcattcaagtacttcgacaccaccatcgaccggaaacgctatacct
ccaccaaagaagtgctggacgccaccctcatccaccagagcatcaccggactttacgaaactcggattgacctctcacagctcggag
gggatggtggcggaggctcgccaaaaaagaagagaaaggtagacccaaagaaaaaacgaaaagtagatccgaaaaagaaga
ggaaggtgggatccctgcccacctgcagctgtcttgatcgagttatacaaaaagacaaaggcccatattatacacaccttgggcagg
accaagtgttgctgctgtcagggaaatcatggagaataggtatggtcaaaaaggaaacgcaataaggatagaaatagtagtgtacac
cggtaaagaagggaaaagctctcatgggtgtccaattgctaagtgggttttaagaagaagcagtgatgaagaaaaagttctttgtttggtc
cggcagcgtacaggccaccactgtccaactgctgtgatggtggtgctcatcatggtgtgggatggcatccctcttccaatggccgaccgg
ctatacacagagctcacagagaatctaaagtcatacaatgggcaccctaccgacagaagatgcaccctcaatgaaaatcgtacctgta
catgtcaaggaattgatccagagacttgtggagcttcattctcttttggctgttcatggagtatgtactttaatggctgtaagtttggtagaagcc
caagccccagaagatttagaattgatccaagctctcccttacatgaaaaaaaccttgaagataacttacagagtttggctacacgattag
ctccaatttataagcagtatgctccagtagcttaccaaaatcaggtggaatatgaaaatgttgcccgagaatgtcggcttggcagcaagg
aaggtcgacccttctctggggtcactgcttgcctggacttctgtgctcatccccacagggacattcacaacatgaataatggaagcactgt
ggtttgtaccttaactcgagaagataaccgctctttgggtgttattcctcaagatgagcagctccatgtgctacctctttataagctttcagaca
cagatgagtttggctccaaggaaggaatggaagccaagatcaaatctggggccatcgaggtcctggcaccccgccgcaaaaaaag
aacgtgtttcactcagcctgttccccgttctggaaagaagagggctgcgatgatgacagaggttcttgcacataagataagggcagtgga
aaagaaacctattccccgaatcaagcggaagaataactcaacaacaacaaacaacagtaagccttcgtcactgccaaccttaggga
gtaacactgagaccgtgcaacctgaagtaaaaagtgaaaccgaaccccattttatcttaaaaagttcagacaacactaaaacttattcg
ctgatgccatccgctcctcacccagtgaaagaggcatctccaggcttctcctggtccccgaagactgcttcagccacaccagctccactg
aagaatgacgcaacagcctcatgcgggttttcagaaagaagcagcactccccactgtacgatgccttcgggaagactcagtggtgcca
atgctgcagctgctgatggccctggcatttcacagcttggcgaagtggctcctctccccaccctgtctgctcctgtgatggagcccctcatta
attctgagccttccactggtgtgactgagccgctaacgcctcatcagccaaaccaccagccctccttcctcacctctcctcaagaccttgcc
tcttctccaatggaagaagatgagcagcattctgaagcagatgagcctccatcagacgaacccctatctgatgacccccctgtcacctgct
gaggagaaattgccccacattgatgagtattggtcagacagtgagcacatctttttggatgcaaatattggtggggtggccatcgcacctg
ctcacggctcggttttgattgagtgtgcccggcgagagctgcacgctaccactcctgttgagcaccccaaccgtaatcatccaacccgcct
ctcccttgtcttttaccagcacaaaaacctaaataagccccaacatggttttgaactaaacaagattaagtttgaggctaaagaagctaag
aataagaaaatgaaggcctcagagcaaaaagaccaggcagctaatgaaggtccagaacagtcctctgaagtaaatgaattgaacc
aaattccttctcataaagcattaacattaacccatgacaatgttgtcaccgtgtccccttatgctctcacacacgttgcggggccctataacc
attgggtcgagggcagaggaagtctgctaacatgcggtgacgtcgaggagaatcctggcccagtgagcaagggcgaggagctgttc
accggggtggtgcccatcctggtcgagctggacggcgacgtaaacggccacaagttcagcgtgtccggcgagggcgagggcgatgc
cacctacggcaagctgaccctgaagttcatctgcaccaccggcaagctgcccgtgccctggcccaccctcgtgaccaccctgacctac
ggcgtgcagtgcttcagccgctaccccgaccacatgaagcagcacgacttcttcaagtccgccatgcccgaaggctacgtccaggag

cgcaccatcttcttcaaggacgacggcaactacaagacccgcgccgaggtgaagttcgagggcgacaccctggtgaaccgcatcga
gctgaagggcatcgacttcaaggaggacggcaacatcctggggcacaagctggagtacaactacaacagccacaacgtctatatcat
ggccgacaagcagaagaacggcatcaaggtgaacttcaagatccgccacaacatcgaggacggcagcgtgcagctcgccgacca
ctaccagcagaacacccccatcggcgacggccccgtgctgctgcccgacaaccactacctgagcacccagtccgccctgagcaaag
accccaacgagaagcgcgatcacatggtcctgctggagttcgtgaccgccgccgggatcactctcggcatggacgagctgtacaagg
aattcaattctaactaggaattcgatatcaagcttatcgataatcaacctctggattacaaaatttgtgaaagattgactggtattcttaactat
gttgctccttttacgctatgtggatacgctgctttaatgcctttgtatcatgctattgcttcccgtatggctttcattttctcctccttgtataaatcctgg
ttgctgtctctttatgaggagttgtggcccgttgtcaggcaacgtggcgtggtgtgcactgtgtttgctgacgcaacccccactggttggggc
attgccaccacctgtcagctcctttccgggactttcgctttccccctccctattgccacggcggaactcatcgccgcctgccttgcccgctgct
ggacaggggctcggctgttgggcactgacaattccgtggtgttgtcggggaaatcatcgtcctttccttggctgctcgcctgtgttgccacct
ggattctgcgcgggacgtccttctgctacgtcccttcggccctcaatccagcggaccttccttcccgcggcctgctgccggctctgcggcct
cttccgcgtcttcgccttcgccctcagacgagtcggatctccctttgggccgcctccccgcatcgataccgtcgacctcgagacctagaaa
aacatggagcaatcacaagtagcaatacagcagctaccaatgctgattgtgcctggctagaagcacaagaggaggaggaggtgggt
tttccagtcacacctcaggtacctttaagaccaatgacttacaaggcagctgtagatcttagccactttttaaaagaaaaggggggactgg
aagggctaattcactcccaacgaagacaagatatccttgatctgtggatctaccacacacaaggctacttccctgattggcagaactaca
caccagggccagggatcagatatccactgacctttggatggtgctacaagctagtaccagttgagcaagagaaggtagaagaagcca
atgaaggagagaacacccgcttgttacaccctgtgagcctgcatgggatggatgacccggagagagaagtattagagtggaggtttga
cagccgcctagcatttcatcacatggcccgagagctgcatccggactgtactgggtctctctggttagaccagatctgagcctgggagctc
tctggctaactagggaacccactgcttaagcctcaataaagcttgccttgagtgcttcaagtagtgtgtgcccgtctgttgtgtgactctggta
actagagatccctcagacccttttagtcagtgtggaaaatctctagcagggcccgtttaaacccgctgatcagcctcgactgtgccttctag
ttgccagccatctgttgtttgcccctcccccgtgccttccttgaccctggaaggtgccactcccactgtcctttcctaataaaatgaggaaatt
gcatcgcattgtctgagtaggtgtcattctattctggggggtggggtggggcaggacagcaagggggaggattgggaagacaatagca
ggcatgctggggatgcggtgggctctatggcttctgaggcggaaagaaccagctggggctctagggggtatccccacgcgccctgtag
cggcgcattaagcgcggcgggtgtggtggttacgcgcagcgtgaccgctacacttgccagcgccctagcgcccgctcctttcgctttcttc
ccttcctttctcgccacgttcgccggctttccccgtcaagctctaaatcgggggctccctttagggttccgatttagtgctttacggcacctcga
ccccaaaaaacttgattagggtgatggttcacgtagtgggccatcgccctgatagacggtttttcgccctttgacgttggagtccacgttcttt
aatagtggactcttgttccaaactggaacaacactcaaccctatctcggtctattcttttgatttataagggattttgccgatttcggcctattggt
taaaaaatgagctgatttaacaaaaatttaacgcgaattaattctgtggaatgtgtgtcagttagggtgtggaaagtccccaggctcccca
gcaggcagaagtatgcaaagcatgcatctcaattagtcagcaaccatagtcccgcccctaactccgcccatcccgcccctaactccgc
ccagttccgcccattctccgccccatggctgactaattttttttatttatgcagaggccgaggccgcctctgcctctgagctattccagaagta
gtgaggaggcttttttggaggcctaggcttttgcaaaaagctcccgggagcttgtatatccattttcggatctgatcagcacgtgttgacaatt
aatcatcggcatagtatatcggcatagtataatacgacaaggtgaggaactaaaccatggccaagttgaccagtgccgttccggtgctc
accgcgcgcgacgtcgccggagcggtcgagttctggaccgaccggctcgggttctcccgggacttcgtggaggacgacttcgccggtg
tggtccgggacgacgtgaccctgttcatcagcgcggtccaggaccaggtggtgccggacaacaccctggcctgggtgtgggtgcgcg
gcctggacgagctgtacgccgagtggtcggaggtcgtgtccacgaacttccgggacgcctccgggccggccatgaccgagatcggcg
agcagccgtgggggcgggagttcgccctgcgcgacccggccggcaactgcgtgcacttcgtggccgaggagcaggactgacacgt
gctacgagatttcgattccaccgccgccttctatgaaaggttgggcttcggaatcgttttccgggacgccggctggatgatcctccagcgc
ggggatctcatgctggagttcttcgcccaccccaacttgtttattgcagcttataatggttacaaataaagcaatagcatcacaaatttcaca
aataaagcatttttttcactgcattctagttgtggtttgtccaaactcatcaatgtatcttatcatgtctgtataccgtcgacctctagctagagctt
ggcgtaatcatggtcatagctgtttcctgtgtgaaattgttatccgctcacaattccacacaacatacgagccggaagcataaagtgtaaa
gcc

P3-pKLV2-U6gRNA(BbsI)-PGKpuro2A-mCherry

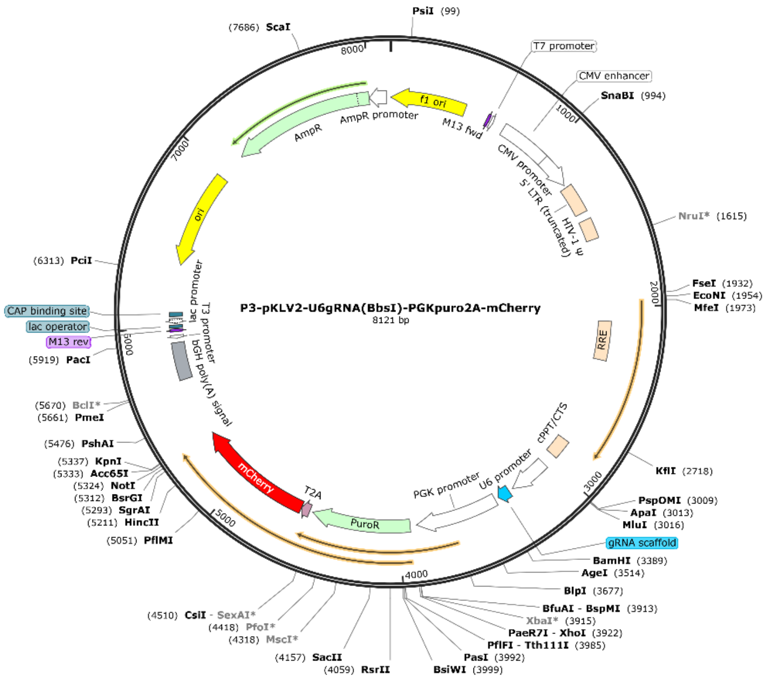

ctaaattgtaagcgttaatattttgttaaaattcgcgttaaatttttgttaaatcagctcattttttaaccaataggccgaaatcggcaaaatccct
tataaatcaaaagaatagaccgagatagggttgagtgttgttccagtttggaacaagagtccactattaaagaacgtggactccaacgtc
aaagggcgaaaaaccgtctatcagggcgatggcccactacgtgaaccatcaccctaatcaagttttttggggtcgaggtgccgtaaag
cactaaatcggaaccctaaagggagcccccgatttagagcttgacggggaaagccggcgaacgtggcgagaaaggaagggaag
aaagcgaaaggagcgggcgctagggcgctggcaagtgtagcggtcacgctgcgcgtaaccaccacacccgccgcgcttaatgcgc
cgctacagggcgcgtcccattcgccattcaggctgcgcaactgttgggaagggcgatcggtgcgggcctcttcgctattacgccagctgg
cgaaagggggatgtgctgcaaggcgattaagttgggtaacgccagggttttcccagtcacgacgttgtaaaacgacggccagtgagcg
cgcgtaatacgactcactatagggcgaattgactagttattaatagtaatcaattacggggtcattagttcatagcccatatatggagttccg
cgttacataacttacggtaaatggcccgcctggctgaccgcccaacgacccccgcccattgacgtcaataatgacgtatgttcccatagt
aacgccaatagggactttccattgacgtcaatgggtggagtatttacggtaaactgcccacttggcagtacatcaagtgtatcatatgcca
agtacgccccctattgacgtcaatgacggtaaatggcccgcctggcattatgcccagtacatgaccttatgggactttcctacttggcagta
catctacgtattagtcatcgctattaccatggtgatgcggttttggcagtacatcaatgggcgtggatagcggtttgactcacggggatttcca
agtctccaccccattgacgtcaatgggagtttgttttggcaccaaaatcaacgggactttccaaaatgtcgtaacaactccgccccattga
cgcaaatgggcggtaggcgtgtacggtgggaggtctatataagcagcgcgttttgcctgtactgggtctctctggttagaccagatctgag
cctgggagctctctggctaactagggaacccactgcttaagcctcaataaagcttgccttgagtgcttcaagtagtgtgtgcccgtctgttgt
gtgactctggtaactagagatccctcagacccttttagtcagtgtggaaaatctctagcagtggcgcccgaacagggacttgaaagcga
aagggaaaccagaggagctctctcgacgcaggactcggcttgctgaagcgcgcacggcaagaggcgaggggcggcgactggtga
gtacgccaaaaattttgactagcggaggctagaaggagagagatgggtgcgagagcgtcagtattaagcgggggagaattagatcg
cgatgggaaaaaattcggttaaggccagggggaaagaaaaaatataaattaaaacatatagtatgggcaagcagggagctagaac
gattcgcagttaatcctggcctgttagaaacatcagaaggctgtagacaaatactgggacagctacaaccatcccttcagacaggatca
gaagaacttagatcattatataatacagtagcaaccctctattgtgtgcatcaaaggatagagataaaagacaccaaggaagctttaga
caagatagaggaagagcaaaacaaaagtaagaccaccgcacagcaagcggccggccgcgctgatcttcagacctggaggagga
gatatgagggacaattggagaagtgaattatataaatataaagtagtaaaaattgaaccattaggagtagcacccaccaaggcaaag
agaagagtggtgcagagagaaaaaagagcagtgggaataggagctttgttccttgggttcttgggagcagcaggaagcactatgggc
gcagcgtcaatgacgctgacggtacaggccagacaattattgtctggtatagtgcagcagcagaacaatttgctgagggctattgaggc
gcaacagcatctgttgcaactcacagtctggggcatcaagcagctccaggcaagaatcctggctgtggaaagatacctaaaggatca
acagctcctggggatttggggttgctctggaaaactcatttgcaccactgctgtgccttggaatgctagttggagtaataaatctctggaaca
gatttggaatcacacgacctggatggagtgggacagagaaattaacaattacacaagcttaatacactccttaattgaagaatcgcaaa
accagcaagaaaagaatgaacaagaattattggaattagataaatgggcaagtttgtggaattggtttaacataacaaattggctgtggt
atataaaattattcataatgatagtaggaggcttggtaggtttaagaatagtttttgctgtactttctatagtgaatagagttaggcagggatatt
caccattatcgtttcagacccacctcccaaccccgaggggacccgacaggcccgaaggaatagaagaagaaggtggagagagag
acagagacagatccattcgattagtgaacggatcggcactgcgtgcgccaattctgcagacaaatggcagtattcatccacaattttaaa

agaaaagggggggattgggggtacagtgcaggggaaagaatagtagacataatagcaacagacatacaaactaaagaattacaa
aaacaaattacaaaaattcaaaattttcgggtttattacagggacagcagagatccagtttggttagtaccgggccctacgcgtccaaggt
cgggcaggaagagggcctatttcccatgattccttcatatttgcatatacgatacaaggctgttagagagataattagaattaatttgactgt
aaacacaaagatattagtacaaaatacgtgacgtagaaagtaataatttcttgggtagtttgcagttttaaaattatgttttaaaatggactat
catatgcttaccgtaacttgaaagtatttcgatttcttggctttatatatcttgtggaaaggacgaaacaccgggtcttcgagaagacacgtttt
agagctagaaatagcaagttaaaataaggctagtccgttatcaacttgaaaaagtggcaccgagtcggtgcttttttggatccaattctac
cgggtaggggaggcgcttttcccaaggcagtctggagcatgcgctttagcagccccgctgggcacttggcgctacacaagtggcctctg
gcctcgcacacattccacatccaccggtaggcgccaaccggctccgttctttggtggccccttcgcgccaccttctactcctcccctagtca
ggaagttcccccccgccccgcagctcgcgtcgtgcaggacgtgacaaatggaagtagcacgtctcactagtctcgtgcagatggacag
caccgctgagcaatggaagcgggtaggcctttggggcagcggccaatagcagctttgctccttcgctttctgggctcagaggctgggaa
ggggtgggtccgggggcgggctcaggggcgggctcaggggcggggcgggcgcccgaaggtcctccggaggcccggcattctgca
cgcttcaaaagcgcacgtctgccgcgctgttctcctcttcctcatctccgggcctttcgacctgcatccatctagatctcgagcagctgaagc
ttaccatgaccgagtacaagcccacggtgcgcctcgccacccgcgacgacgtccccagggccgtacgcaccctcgccgccgcgttcg
ccgactaccccgccacgcgccacaccgtcgatccggaccgccacatcgagcgggtcaccgagctgcaagaactcttcctcacgcgc
gtcgggctcgacatcggcaaggtgtgggtcgcggacgacggcgccgcggtggcggtctggaccacgccggagagcgtcgaagcgg
gggcggtgttcgccgagatcggcccgcgcatggccgagttgagcggttcccggctggccgcgcagcaacagatggaaggcctcctg
gcgccgcaccggcccaaggagcccgcgtggttcctggccaccgtcggcgtctcgcccgaccaccagggcaagggtctgggcagcg
ccgtcgtgctccccggagtggaggcggccgagcgcgccggggtgcccgccttcctggagacctccgcgccccgcaacctccccttcta
cgagcggctcggcttcaccgtcaccgccgacgtcgaggtgcccgaaggaccgcgcacctggtgcatgacccgcaagcccggtgccg
gcggcgggtccggaggagagggcagaggaagtctcctaacatgcggtgacgtggaggagaatcctggcccaatggtgagcaagg
gcgaggaggataactccgccatcatcaaggagttcctgcgcttcaaggtgcacatggagggctccgtgaacggccacgagttcgagat
cgagggcgagggcgagggccgcccctacgagggcacccagaccgccaagctgaaggtgaccaagggtggccccctgcccttcgc
ctgggacatcctgtcccctcagttcatgtacggctccaaggcctacgtgaagcaccccgccgacatccccgactacttgaagctgtccttc
cccgagggcttcaagtgggagcgcgtgatgaacttcgaggacggcggcgtggtgaccgtgacccaggactcctctctgcaggacggc
gagttcatctacaaggtgaagctgcgcggcaccaacttcccctccgacggccccgtaatgcagaagaaaaccatgggctgggaggc
ctcctccgagcggatgtaccccgaggacggcgccctgaagggcgagatcaagcagaggctgaagctgaaggacggcggccacta
cgacgctgaggtcaagaccacctacaaggccaagaagcccgtgcagctgcccggcgcctacaacgtcaacatcaagttggacatca
cctcccacaacgaggactacaccatcgtggaacagtacgaacgcgccgagggccgccactccaccggcggcatggacgagctgta
caagtgagcggccgctaggtacctttaagaccaatgacttacaaggcagctgtagatcttagccactttttaaaagaaaaggggggact
ggaagggctaattcactcccaaagaagtcaagatctgctttttgcctgtactgggtctctctggttagaccagagtctctctggttagaccag
atctgagcctgggagctctctggctaactagggaacccactgcttaagcctcaataaagcttgccttgagtgcttcaagtagtgtgtgcccg
tctgttgtgtgactctggtaactagagatccctcagacccttttagtcagtgtggaaaatctctagcagtttaaacccgctgatcagcctcgac
tgtgccttctagttgccagccatctgttgtttgcccctcccccgtgccttccttgaccctggaaggtgccactcccactgtcctttcctaataaa
tgaggaaattgcatcgcattgtctgagtaggtgtcattctattctggggggtggggtggggcaggacagcaagggggaggattgggaag
tcaatagcaggcatgctggggatgcggtgggctctatggcggccgttaattaagcttttgttccctttagtgagggttaattgcgcgcttggc
gtaatcatggtcatagctgtttcctgtgtgaaattgttatccgctcacaattccacacaacatacgagccggaagcataaagtgtaaagcct
ggggtgcctaatgagtgagctaactcacattaattgcgttgcgctcactgcccgctttccagtcgggaaacctgtcgtgccagctgcattaa
tgaatcggccaacgcgcggggagaggcggtttgcgtattgggcgctcttccgcttcctcgctcactgactcgctgcgctcggtcgttcggct
gcggcgagcggtatcagctcactcaaaggcggtaatacggttatccacagaatcaggggataacgcaggaaagaacatgtgagca
aaaggccagcaaaaggccaggaaccgtaaaaaggccgcgttgctggcgtttttccataggctccgcccccctgacgagcatcacaa
aaatcgacgctcaagtcagaggtggcgaaacccgacaggactataaagataccaggcgtttccccctggaagctccctcgtgcgctct
cctgttccgaccctgccgcttaccggatacctgtccgcctttctcccttcgggaagcgtggcgctttctcatagctcacgctgtaggtatctca
gttcggtgtaggtcgttcgctccaagctgggctgtgtgcacgaaccccccgttcagcccgaccgctgcgccttatccggtaactatcgtctt
gagtccaacccggtaagacacgacttatcgccactggcagcagccactggtaacaggattagcagagcgaggtatgtaggcggtgct
acagagttcttgaagtggtggcctaactacggctacactagaaggacagtatttggtatctgcgctctgctgaagccagttaccttcggaa
aaagagttggtagctcttgatccggcaaacaaaccaccgctggtagcggtggtttttttgtttgcaagcagcagattacgcgcagaaaaa
aaggatctcaagaagatcctttgatcttttctacggggtctgacgctcagtggaacgaaaactcacgttaagggattttggtcatgagattat
caaaaaggatcttcacctagatccttttaaattaaaaatgaagttttaaatcaatctaaagtatatatgagtaaacttggtctgacagttacca
atgcttaatcagtgaggcacctatctcagcgatctgtctatttcgttcatccatagttgcctgactccccgtcgtgtagataactacgatacgg
gagggcttaccatctggccccagtgctgcaatgataccgcgagacccacgctcaccggctccagatttatcagcaataaaccagccag
ccggaagggccgagcgcagaagtggtcctgcaactttatccgcctccatccagtctattaattgttgccgggaagctagagtaagtagttc
gccagttaatagtttgcgcaacgttgttgccattgctacaggcatcgtggtgtcacgctcgtcgtttggtatggcttcattcagctccggttccc
aacgatcaaggcgagttacatgatcccccatgttgtgcaaaaaagcggttagctccttcggtcctccgatcgttgtcagaagtaagttggc
cgcagtgttatcactcatggttatggcagcactgcataattctcttactgtcatgccatccgtaagatgcttttctgtgactggtgagtactcaac
caagtcattctgagaatagtgtatgcggcgaccgagttgctcttgcccggcgtcaatacgggataataccgcgccacatagcagaacttt
aaaagtgctcatcattggaaaacgttcttcggggcgaaaactctcaaggatcttaccgctgttgagatccagttcgatgtaacccactcgt

gcacccaactgatcttcagcatcttttactttcaccagcgtttctgggtgagcaaaaacaggaaggcaaaatgccgcaaaaaagggaat aagggcgacacggaaatgttgaatactcatactcttcctttttcaatattattgaagcatttatcagggttattgtctcatgagcggatacatatt tgaatgtatttagaaaaataaacaaataggggttccgcgcacatttccccgaaaagtgccac

Supplementary Fig. 8. Sequence and map of the plasmids used for DNA methylation editing. More information and the plasmids will be available upon request. Plasmids constructed for this study can be obtained via Addgene.

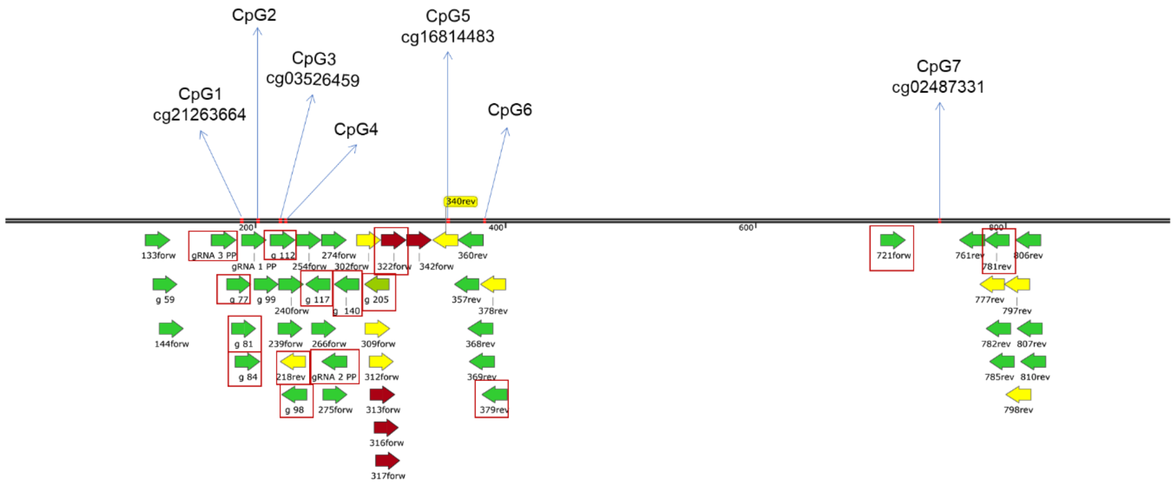

Supplementary Fig. 9. Map of the gRNAs designed to target the 1q21.1 DMR.
All the gRNAs were designed by CRISPOR Version 4.98. The gRNAs in the rectangle were used for the gRNA screening on HEK293T cells (the sequences are listed in the Supplementary Table 10).

Supplementary Methods

Ethics approval and consent to participate

All experiments on human subjects were approved by the Regional Ethical Review Board in Stockholm and carried out in accordance with institutional guidelines. Written informed consent was obtained from all study participants. iPSC experiments are in accordance with an IRB approval from the Ethical Review Boards in Stockholm, Sweden. Zebrafish experiments were carried out according to the guidelines of the Ethics Committee of IGBMC and ethical approval was obtained from the French Ministry of Higher Education and Research under the number APAFIS#15025-2018041616344504.

Cohorts

Details of the cohorts are described in the Supplementary Table 1. Briefly, for genome-wide DNA methylation and methylation quantitative trait locus (meQTL) analysis used in cohort 1, peripheral blood samples were collected form 140 MS, patients including 120 RRMS, 4 PPMS and 16 SPMS patients, and 139 healthy individuals (HC), as previously described [5]. An independent cohort, cohort 2, consisting of 48 RRMS and 36 PPMS patients (matched for age, sex, disease duration and Swedish descent) was used for pyrosequencing validation and locus-specific meQTL analysis [6]. Cohort 3 and the additional Italian cohort used for genetic association study comprises 1,104 PPMS and 11,335 BOMS, as described in the corresponding sections below. Gene expression data (RNA-sequencing) from bulk brain tissue samples of progressive MS patients (5 PPMS and 7 SPMS) and non-neurological controls (n = 10), previously described [7], were used for correlation network analysis and validation was performed in neuronal snRNA-seq data [8]. Additionally, we utilized publicly available databases from xQTL serve [9] (n = 543 bulk prefrontal cortex samples, http://mostafavilab.stat.ubc.ca/xQTLServe/) and GTEx (selecting all available nervous tissues, https://gtexportal.org/) platforms to address meQTL and eQTL effects in the CNS. No sample size calculation was performed for the cohorts involved in this study.

Genome-wide DNA methylation and meQTL analyses (cohort 1)

DNA methylation analysis. The methylation data from Infinium HumanMethylation450 (450K) arrays was preprocessed as previously described [5] using the Illumina default procedure implemented in the Bioconductor minfi package [10]. Briefly, all samples were normalized together using the minfi preprocessQuantile function. The probe level raw data for each sample were normalized using Illumina's control probe scaling procedure and converted to methylation β values on the 0–1 scale (M/(M + U + 100), where M and U represent the methylated and unmethylated signal intensities, respectively). Cell counts for the six major cell types in blood (granulocytes, B cells, $CD4^+$ T cells, $CD8^+$ T cells, monocytes, and NK cells) for each individual were estimated using the estimateCellCounts function in minfi package [10], which obtain sample-specific estimates of cell proportions based on reference information on cell-specific methylation signatures [11]. Results from the estimation can be found in [5]. To identify differentially methylated regions (DMRs) associated with the PPMS phenotype, we used the bumphunter function in minfi package [10] with adjustment for confounders: age, sex, self-reported smoking status (ever smokers vs. never smokers), hybridization date, and the first two principle components of estimated differential cell counts. Region that has a family wise error rate (FWER) less than 0.05 with 1000 resamples and contains at least 2 probes was identified as a trait-associated DMR.

Methylation QTL analysis. To identify potential genetic dependency, the PPMS-associated DMR was tested for association with genotype (594,262 SNPs) using an additive minor-allele dosage model. Genotype-DMR associations were corrected for multiple testing using a stringent Bonferroni-adjusted threshold of 0.05.

Locus-specific DNA methylation and meQTL analyses (cohort 2)

DNA methylation analysis. For validation of the identified PPMS-associated DMR, pyrosequencing analysis was performed using 500 ng of genomic DNA samples previously converted to bisulfite DNA (BS-DNA, EZ DNA methylation kit, ZYMO research) with PyroMark Q96 system (Qiagen). Primers and probes for three sequencing assays covering 7 CpG sites in the locus were designed by PyroMark Design software (Qiagen) (Supplementary Table 12, Supplementary Fig. 5). Around 10 ng of BS-DNA was amplified using PyroMark PCR kit (Qiagen) and the forward and 5′-biotinylated reverse primers. The entire PCR product, 4 pmol of the sequencing probe and streptavidin sepharose high-performance beads (GR Healthcare) were used for pyrosequencing on a PyroMark Q96 ID pyrosequencing instrument (Qiagen) using the PyroMark Gold 96 Reagent kit (Qiagen). Methylation levels were determined by the ratio of C and T by the PyroMark CpG software 1.0.11 (Qiagen) and expressed as percentage methylation at each CpG site. To verify the efficiency and sensitivity of the PCR-pyrosequencing, we used standard curves with unmethylated and methylated human BS-DNA samples (Qiagen). To test the differences in DNA methylation between PPMS and RRMS patients for each CpG site, non-parametric Mann-Whitney U test was applied with GraphPad Prism software (PRISM 7.0; GraphPAD Software Inc., San Diego, CA, USA). We have done extensive SNP and sequence analyses to assure that the methylation measurements of the majority of the DMR CpGs are not a result of the technical measurement artefacts driven by a potential effect of SNPs on the CpGs [12,13], pyrosequencing or 450K assays.

Methylation QTL analysis. Methylation data was RANK transformed in R using R Core team (Vienna, Austria, https://www.R-project.org/). Genotyping was carried out at deCODE (deCODE genetics/Amgen, Reykjavik, Iceland) using Illumina OmniExpress chip with 716,503 SNPs mapped to the Human Assembly Feb.2009 (GRCh37/hg19). Of 84 individuals, 83 where genotyped in deCODE and 82 of them passed QC. We performed meQTL analysis of chromosome 1 from bp 146500000 to bp 147000000, in PLINK [14] excluding SNPs with less than 98% genotyping rate and SNPs that were not in Hardy-Weinberg equilibrium ($p < 0.05$) and corrected for 5 population based (ancestral informative markers) principal component analysis covariates. After quality control, 123 SNPs remained in the region. Genotype-CpG associations were corrected for multiple testing using stringent Bonferroni-adjusted threshold of 0.05.

Genetic association study in the Swedish (SWE) cohort

Patients from the Swedish (SWE) cohort were genotyped in two different batches at deCODE Genetics using Illumina Human OmniExpress 24 v1 (OE) and Global Screening Array MD 24 v2 (GSA) arrays, following manufacturer’s instructions. The cohort was aligned to the forward strand of the hg19 reference genome based on strand information from the Illumina array manifest files. Samples with less than 95% genotyping yield were excluded. Samples with a mismatch between reported and genetic sex were also excluded using a linkage disequilibrium (LD) pruned set of high-quality chromosome X variants. Genetic variants were filtered based on several criteria, including genotype missingness, Hardy-Weinberg Equilibrium (HWE), minor allele frequency (MAF), and differential missingness between cases and controls. Palindromic variants were also filtered based on alternate allele frequency. Individuals with an absolute inbreeding coefficient greater than 0.05 or relatedness at the third degree or closer were excluded. To control for population stratification prior to imputation, Principal Components (PC) were calculated, and outliers were excluded. The Haplotype Reference Consortium (HRC; version 1.1) imputation reference panel 8 was used for phasing and imputation of genotyping data [15]. A total of 7,682,164 autosomal variants passed quality controls.

Starting from these, for the genetic association analysis we extracted all the imputed SNPs (n = 3,057) in the extended chr1 locus (from bp 146500000 to bp 147000000). A Principal Component Analysis (PCA) was performed using whole-genome autosomal markers after LD pruning (100 bp window size, 2 bp step, pairwise r2 threshold of 0.1). The first 8 PC and biological sex were included as covariates in the generalized linear model analysis of SNP-to-phenotype association (PPMS = 603 versus BOMS = 9,247, to account for residual population stratification. The PCA and the association analyses were run separately for individuals genotyped on OE chip and those

genotyped on GSA arrays, to minimize the batch effect exerted by array architecture and potential differences in recruitment of individuals between the two datasets. Subsequently, the results from the two independent association studies were meta-analyzed using fixed-effect and random-effect models as implemented in PLINK. To gain an insight on the underlying genetic structure, we estimated the haplotype blocks in the extended chr1 locus using the largest cohort (OE cohort) as reference on SNPs with MAF => 0.05 adopting the standard method integrated in PLINK [16]. Sixty-nine LD blocks were identified and used in a Bonferroni correction to account for multiple testing.

Genetic association study in the Italian (ITA) cohort

For the Italian cohort, patients were recruited at the Laboratory of Human Genetics of Neurological Disorders at the San Raffaele Scientific Institute in Milan, Italy and genotyped on Illumina platforms. Prior to imputation, we excluded subjects for which sex mismatch, those with call-rate < 90% and outliers exceeding the mean level of heterozygosity by > 3 standard deviations. At variant level, we discarded rare SNPs with MAF < 1%, SNPs with a call-rate < 90% and those departing from HWE at $p < 10 \times 10^{-6}$. Imputation was carried out to HRC reference genome [15]. A logistic regression model, as described for the SWE cohort, was used to study the association between the SNPs in the extended chr1 locus and the course of MS in a total of 2,589 patients (PP = 501; BOMS = 2,088). Sex and PC 1 to 8 were used as covariates in the model.

SWE and ITA meta-analysis

A fixed-effect model meta-analysis of the standard errors of the odds ratio, as implemented in Plink [17], was applied on the three cohorts. The number of common variants in all the cohorts was 2,676. Multiple testing issue was addressed as described for the SWE cohort.

In-vitro methylation assay

To address the regulatory features of the identified DMR, we used in-vitro DNA methylation reporter assay. A 927 bp fragment encompassing the identified DMR was amplified using primers containing overhanging *SpeI* and *NsiI* restriction sites (Supplementary Table 12). We used blood genomic DNA from PPMS patients presenting with low (rs1969869: CC) and high (rs1969869: AA) methylation levels at the identified DMR. The amplified products in direct and reverse orientation were inserted into pCpG-free promoter vector (Invivogen) containing a Lucia luciferase reporter and into a pCpG-free basic vector (Invivogen) containing a murine secreted embryonic alkaline phosphatase (mSEAP) reporter gene for assessment of enhancer and promoter activity, respectively. As the body of these vectors is devoid of any CpGs, any impact of DNA methylation on reporter gene expression is restricted to the inserted fragment only. All the constructs were either completely methylated (57 CpGs) using *M.SssI* or partially methylated (7 CpGs residing in the GCGC sequence) by *HhaI* methyltransferases (New England BioLabs) using 1 ug of the vectors and 1 unit of the enzymes. The mock methylated control was treated equally but in absence of any methyltransferases and corresponds to unmethylated inserts. After the purification of the methylated, partially- and mock-methylated constructs (QIAquick PCR purification Kit, Qiagen), the efficiency of methylation was assessed using an EpiJET DNA Methylation analysis Kit (*MspI*/*HpaII*) (ThermoFisher Scientific), followed by gel electrophoresis (Supplementary Fig. 6). Original vectors treated by *M.SssI* and *HhaI* or mock-treated were used as controls. Human embryonic kidney HEK293T cells were cultured in Dulbecco's Modified Eagle's medium in 96 well plates and co-transfected with 90 ng of the Lucia or SEAP constructs and 5 ng of the control vector pGL4-TK-hH Luc constitutively expressing Renilla luciferase, using Lipofectamine 3000 Transfection Reagent (Qiagen). Approximately, 48 hours post transfection, Lucia, SEAP and Renilla activities were measured using QUANTI-Luc (Invivogen), the Phospha-Light System (Applied Biosystems) and the Dual-Glo Luciferase Assay System (Promega), respectively, according to manufacturer's instructions, on the GloMax 96 Microplate Luminometer (Promega). Both direct and reverse orientations of the sequence were tested. Lucia or SEAP signals were normalized against Renilla (triplicate) and experiments were replicated at least two times.

CRISPR/dCas9-TET1 epigenome editing

dCas9-TET1 and gRNA generation. Details and maps of the final constructs used in this study are presented in Supplementary Figure 7. Briefly, we engineered a P3-dCas9-Tet1-GFP-Puro (Addgene #190728) construct as follows. First, to be able to express the gRNAs from the same vector, we mutated the *BbsI* sites in the TET1 sequence (without changing the protein sequence) synthesized by Eurofins (Eurofins MWG Operon Ebersberg, Germany). We then utilized the backbone of a pdCas9-DNMT3A-EGFP plasmid (Addgene #71666) [18] and replaced DNMT3A with TET1 sequence. This cassette was previously engineered to express the original EGFP sequence in a double reporter cassette containing EGFP-T2A-Puromycin under the control of an independent CMV promoter to allow sufficient expression of GFP signal for post-transfection cell sorting. We proceeded similarly with the TET1-IM construct which expresses a deactivated TET1 catalytic unit. The final plasmid expressed dCas9-TET1 (or dCas9-TET1-IM) and CMV-EGFP-T2A-Puromycin double marker unit. All gRNAs were designed by CRISPOR Version 4.98 [19] both on the sense and antisense strands, with sequence and mapping presented in the Supplementary Table 12 and Supplementary Fig. 8.

To address dCas9-TET1-mediated epigenome editing in SH-SY5Y cell line, we utilized the lentivirus version of the cassettes. We used Fuw-dCas9-Tet1CD (Addgene #84475) and Fuw-dCas9-Tet1CD_IM (Addgene #84479) plasmids and added the EGFP marker to facilitate sorting of positively transduced cells (new plasmids named as P3-Lenti-dCas9-Tet1-GFP (Addgene #190729) and P3-Lenti-dCas9-Tet1IM-GFP. We used pKLV2-U6gRNA3(BbsI)-PGKpuro2ABFP (Addgene #67990) to express the gRNA and replaced the BFP with mCherry (named as P3-pKLV2-U6gRNA(BbsI)-PGKpuro2A-mCherry). All constructs were confirmed by Sanger sequencing (KiGene) and chromatograms were analyzed using SnapGene software 5.2.3 (GSLBiotech). Plasmids constructed for this study can be obtained from Addgene.

dCas9-TET1 delivery to HEK293T and SH-SY5Y cells. To test the efficiency of epigenome editing, we exploited HEK293T ease of transfection and performed gRNAs screen. Different gRNAs were transfected either individually or in combination based on the target site, using Lipofectamine 3000 (Invitrogen). For all experiments both on HEK293T and SH-SY5Y cells, DNA was extracted after 72 hours and bisulfite conversion was performed using 200 ng of the extracted DNA (BS-DNA, EZ DNA methylation kit, ZYMO research). DNA methylation was assessed using pyrosequencing, as described above. For all experiments conducted on SH-SY5Y cells, we delivered a mix of the two gRNAs that showed the highest efficiency in reducing methylation in HEK293T cells (gRNA #2 and #3).

For the generation of lentiviruses and subsequent transduction of SH-SY5Y cells, HEK293T cells were seeded (~40-50 % confluent) in 2 ml DMEM with 10% serum and 2 mM glutamine per well in 6-well plates. The next day, cells had reached 60-70 % confluency and were co-transfected with transfer plasmids as P3-Lenti-dCas9-Tet1-GFP (Addgene #190729) or P3-Lenti-dCas9-Tet1IM-GFP, as well as P3-pKLV2-U6gRNA(BbsI)-PGKpuro2A-mCherry, psPAX2 (Addgene #12260) and pMD2.G (Addgene #12259) using Lipofectamine 3000 (Invitrogen) transfection reagent as per manufacturer instructions. After overnight incubation, medium was removed, and fresh medium added containing 5% serum and 1X Pen-strep. Cells were further incubated for 48h and 72h post-transfection and the virus containing supernatant collected and centrifuged for 10 mins at 500g to remove any cell debris. The supernatant was concentrated using Lentivirus Precipitation Solution (cat. #VC100; AlStem) according to the manufacturer’s instructions and used to transduce SH-SY5Y cells. SH-SY5Y cell line was transduced with virus in complete medium (DMEM with 10% serum and 1X Pen-Strep) containing 8 µg/ml Polybrene (Sigma) with spin infection. Medium was changed 3-4 times to remove any virus residues and cells were used for further assays.

qPCR analysis

Total RNA and DNA were extracted using AllPrep DNA/RNA Kits (Qiagen) according to the manufacture instruction. RNA and DNA concentrations and quality were verified by QIAxpert (Qiagen). Reverse transcription of RNA was performed using the manufacturer's instructions of

iScript™ cDNA Synthesis Kit (Bio-Rad Laboratories, Inc., CA) with OligodT and Random Hexamer primers, generating cDNA for subsequent gene expression analysis. Real-time PCR was performed on a BioRad CFX384 Touch Real-Time PCR Detection System using iQ™ SYBR® Green Supermix (Bio-Rad Laboratories, Inc., CA) in a three step PCR: 95 °C:3 min, followed by 40 cycles of 95 °C:10 s, 60 °C:30 s and 72 °C:30 s. The relative expressions of the selected genes were normalized to the reference gene *GAPDH*. The specificity of real time PCR reaction was verified by the melt curve analysis. The expression level of selected genes were analyzed using ΔΔCT method [20] and compared via independent t-test. All statistical analyses were performed in GraphPad Prism 6 and 7 (GraphPad Software).

Correlation network analysis in MS brain

Raw data analysis. The fastq files corresponding to bulk gene expression (RNA-sequencing) data from brain tissue samples of progressive MS patients ($n_{PPMS} = 5$, $n_{SPMS} = 7$) and non-neurological controls (n = 10) [7,8] were extracted from the RAW RNA sequence files and checked for quality control using multiqc software to make them ready for alignment [21]. After trimming using the trimgalore program [22], fastqc files were aligned and annotated using STAR aligner and Stringtie software [23] by applying human hg38 refseq information from UCSC. The analysis was performed on the extracted count matrix using bash and Python.

Network analysis. In order to utilize a brain-specific network module, we applied a previously established bioinformatic pipeline utilizing co-expression network analysis [24], as briefly described below. The count matrix was loaded into R(3.6.1) environment and quantile normalized using the glimma package [25]. Spearman correlation is applied on every gene pair and permuted for 10000 times to determine which interactions are significant (FDR < 0.05), which avoids biased filtering of the network based on correlation R value. The function also integrates hub connectivity significance for including only the interactions that have significant connectivity in the network. The produced network consisted of 5 million interactions among 27,059 genes, which limits the inherent resolution for defining modules which overlooks the multiscale organization of the network where compact clusters co-exist. In order to overcome this limitation, the correlation network was embedded on a spherical surface, thereby creating a planar maximally filtered network devoid of cross links. The final network consisted of 0.5 million interactions among 27,059 expressed genes from the RNAseq data. The planar maximally filtered graph is then clustered by implementing multiscale clustering algorithm (MCA) from the MEGENA package in R. MCA incorporates three distinct criteria to identify locally coherent clusters while maintaining a globally optimal partition. First, shortest path distances are utilized to optimize within-cluster compactness. Second, local path index is used to optimize local clustering structure. Third, overall modularity is employed to identify optimal partition. The final network clustered into 757 non-overlapping modules.

For the validation data, we applied the same pipeline as described above on several datasets (Supplementary Table 11). In the CUX2^{+} neuronal snRNA-seq count data [8], planar maximal filtration of the Spearmen correlated network of 10780 genes was multiscale clustered using the MEGENA package in R. This resulted in 91 modules out of which 1 module with *CHD1L* was significant and was further analyzed with Fisher enrichment test and pathway analysis using clusterProfiler.

Cluster Trait association analysis. Principal component analysis (PCA) is first performed for each cluster. Next, correlation between the first principal component and each trait was computed as cluster relevance to the trait. The 757 clusters identified from the correlation network were evaluated for the relevance to PPMS, SPMS and control phenotypes. Three clustered passed FDR P-value < 0.05.

Zebrafish *chd1l* knock-out experiments

Zebrafish husbandry. Zebrafish (Danio rerio) were raised and maintained as described in[26]. Adult zebrafish were raised in 15 L tanks containing a maximum of 24 individuals, and under a 14 h- 10 h light-dark cycle. The water had a temperature of 28.5 °C and a conductivity of 200 µS and was continuously renewed. The fish were fed three times a day, with dry food and Artemia salina larvae. Embryos were raised in E3 medium, at 28.5 °C, under constant darkness. The wild type AB strain, the *chd1l*^{sa14029} (TL) mutant line (#15474), carrying the mutation C>T at the genomic location Chr6:36844273 (GRCz11), the *Tg(olig2:EGFP)vu12* (AB) (#15211) line were obtained from the European Zebrafish Resource Center. All fish lines reproduce normally, no skewed sex ratio was observed and chd1l homozygote mutants were recovered in expected Mendelian ratio. All animal experiments were carried out according to the guidelines of the Ethics Committee of IGBMC and ethical approval was obtained from the French Ministry of Higher Education and Research under the number APAFIS#15025-2018041616344504.

Genotyping of the chd1l^{sa14029} *mutant line.* Adult fish were anesthetized in 80 µg/mL tricaine. Fin clips were digested in 50 µL of 50mM NaOH for 15 minutes at 95 °C, and the reaction was neutralized by adding 5 µL of 1M Tris-HCl pH7. The genomic region encompassing the sa19827 mutation was amplified by PCR reaction, using the following primers: 5'-CAGCGTCAGTTTTGCTACCC-3' and 5'-CACCTGGATTGTTCTTGAGC-3'. The PCR product was digested by the Taq α I enzyme, a restriction enzyme whose restriction site is disrupted by the sa14029 mutation. We ran the digestion product on a 2.5% agarose gel for 30 minutes at 135 V. For control *chd1l+/+*, 2 bands are detected (500 base pairs and 150 base pairs); for heterozygous *chd1l*$^{sa14029/+,}$ 3 bands are detected (650 base pairs, 500 base pairs and 150 base pairs); and for homozygous *chd1l*$^{sa14029/sa14029}$ a single 650 base pair-band is detected. In figures and main manuscript, *chd1l+/-* refers to heterozygous *chd1l*$^{sa14029/+}$ and *chd1l-/-* refers to homozygous *chd1l*$^{sa14029/sa14029.}$

Wholemount immunostaining. Larvae were fixed in Dent's fixative (80% methanol, 20% dimethylsulphoxide [DMSO]) overnight at 4°C. The embryos were permeabilized with proteinase K, then postfixed with 4% PFA and washed in PBSTX (PBS.0.5%, Triton X-100). After rehydration in PBS, PFA-fixed embryos were washed in IF buffer (0.1% Tween-20, 1% BSA in PBS 1X) for 10 min at room temperature. The embryos were incubated in the blocking buffer (10% FBS, 1% BSA in PBS 13) for 1 hr at room temperature. After two washes in IF Buffer for 10 min each, embryos were incubated in the first antibody solution, 1:1,000 anti-acetylated tubulin (T7451, Sigma-Aldrich), in blocking solution, overnight at 4_C. After two washes in IF Buffer for 10 min each, embryos were incubated in the secondary antibody solution, 1:1,000 Alexa Fluor goat anti-mouse IgG (A21207, A11001, Invitrogen), in blocking solution, for 1 hr at room temperature. Images were acquired using MacroFluo ORCA Flash (Leica) system. Maximum projection of Z-stacks was used for further analysis using Fiji.

Imaging of the oligodendrocyte lineage. Transgenic *Tg(olig2:EGFP); chd1l+/+* and *Tg(olig2:EGFP); chd1l+/-* larvae were raised up to 3 days at 28.5°C and then fixed for 5 hours using paraformaldehyde 4%. Larvae were bleached for pigment removal for 10 minutes in depigmentation solution (3% H_2O_2/0.5% KOH) and washed 3 times using PBS-Tween 0.1% before imaging. Fish were imaged dorsally for the head and laterally for the spinal cord. Images of the spinal cord were inverted for easier analysis.

Statistical analysis. At least N=3 replicates (one batch of eggs corresponds to one replicate) for each genotype were analyzed. Number of fish per condition is indicated (n). Routh tests were performed using GraphPad Prism v8.0.2.263 for outliers exclusion. When normality and equal variance were validated using Shapiro Test and Bartlett's test, a Student T-test was performed for p-value. If variance were not equal, a Student's T-test with Welch's correction was applied. If normality was not observed, a Wilcoxon T-test was used. GraphPad Prism v8.0.2.263 (GraphPad Software) was used to visualize data as mean ± SEM for at least N=3 replicates per genotype and analysis.

iPSC reprogramming and neural differentiation

Skin biopsies were collected under standardized conditions according to the Regional Ethical Review Boards in Stockholm, Sweden and fibroblast cultures were established, tested negative for mycoplasma and reprogrammed into iPSCs using mRNA reprogramming, as described earlier [27]. iPSC lines were expanded in mTeSR1 media (STEMCELL Technologies) on Matrigel (Corning)-coated plates and purified via magnetic cell sorting (MACS) using Anti-TRA-1-60 MicroBeads (Miltenyi Biotec) according to manufacturer's instructions. Informed consent was obtained from the study participants.

Neural progenitor cells (NPCs) were generated through dual-SMAD pathway inhibition as described earlier. Briefly, 10,000 iPSCs were seeded in V-bottom ultra-low-attachment 96-well plate and cultured for 1 week in embryoid body medium containing Advanced DMEM/F12 (ThermoFisher Scientific) supplemented with dorsomorphin (1 µM, Sigma), N-2 (1X, ThermoFisher Scientific), B27 -RA (1X, ThermoFisher Scientific), and SB431542 (5 µM, Tocris Bioscience). EBs were then transferred to growth factor reduced Matrigel-coated 6-well plates and cultured for 1 week in media containing DMEM/F12 supplemented with N-2 (1X) and Laminin 521 (1 µg/ml, Biolamina) to induce neural rosettes. After 7 days, the rosettes were manually isolated and expanded on growth factor reduced Matrigel-coated 6-well plates in NPC media composed of Neurobasal (ThermoFisher Scientific) and Advanced DMEM/F12 (1:1), supplemented with N-2 (1X), B27 -RA (1X), FGF2 (20 ng/ml, Peprotech), hLIF (10 ng/ml, Peprotech), CHIR99021 (3 µM, R&D Systems), SB431541 (2 µM) and Y-27632 rock inhibitor (10 µM, STEMCELL Technologies). Resulting NPCs were purified with CD271 (Miltenyi Biotec) negative selection followed by CD133 (Miltenyi Biotec) positive selection via MACS.

For neuronal differentiation, NPCs were seeded in either 96- or 24-well plates coated with poly-L-ornithine (10 µg/ml) and laminin (5 µg/ml) and cultured with NPC media for a day. On the following day, media was replaced with week1 differentiation media consisting of Neurobasal and DMEM/F12 (1:1) supplemented with N-2 (1X), B27 (1X), MEM non-essential amino acids (1X, Thermofisher), Glutamax (1X, ThermoFisher Scientific), Penicillin-Streptomycin (1X, ThermoFisher Scientific), BDNF (20 ng/ml, Peprotech), GDNF (10ng/ml, Peprotech), ascorbic acid (200uM, STEMCELL Technologies) and cAMP (1 µM, Sigma). Week 2 onwards, differentiation media was supplemented with 50% BrainPhys (STEMCELL Technologies) and laminin (1 µg/ml) and half-media changes were performed until the end of differentiation (5 weeks).

Human iPSC *CHD1L* knock-down experiments

The Accell human *CHD1L* siRNA SMARTpool and Non-targeting control pool purchased from Dharmacon were freshly supplemented into NPC media or differentiation media at a final concentration of 1 µM and maintained throughout the process of neuronal differentiation.

Immunocytochemistry

For immunofluorescence staining of NPCs and forebrain neurons, cells were fixed with 4% paraformaldehyde (Thermo Fisher Scientific) in Phosphate Buffered Saline (PBS) for 15 min at room temperature. Following washing with PBS, the samples were blocked for 1 h at room temperature in blocking solution, consisting of 3% v/v bovine serum albumin (R&D Systems) in 0.3% Triton X-100 in PBS. The samples were then incubated overnight at 4 °C in primary antibody solution (3% BSA in 0.1% Triton X 100) supplemented with antibodies from the following list: anti-SOX1 (1:200 dilution; 4194, Cell Signaling), anti-SOX2 (1:300 dilution; 14-9811-82, ThermoFisher Scientific), anti-PAX6 (1:100 dilution; AB528427, DHSB), anti-MAP2 (1:500 dilution; ab5392,

Abcam) and anti-SYN1 (1:200 dilution; 106 002, Synaptic Systems). After washing thrice with PBS, the cells were incubated for 2 h at room temperature with appropriate secondary antibodies from this list: Goat anti-chicken Alexa Fluor 647 (1:500 dilution; A21449, ThermoFisher Scientific), Goat anti-mouse Alexa Fluor 488 (1:500 dilution; A28175, ThermoFisher Scientific), Goat anti-rabbit Alexa Fluor 555 (1:500 dilution; A21428, ThermoFisher Scientific). The nuclei were counterstained with DAPI (1:1000 dilution; 62248, ThermoFisher Scientific) and the 27 stained samples were mounted using fluorescence mounting medium (S302380-2, Dako). Images of the stained samples were acquired using an LSM-800 confocal laser scanning microscope with a 20× objective equipped with Zeiss Zen (Blue edition) (v2.3) and fluorescence images were processed and analyzed using ImageJ software (v2.0.0).

Branching analysis was performed using the NeuronJ plugin [28], http://www.imagescience.org/meijering/software/neuronj/) in ImageJ to trace primary neurites, defined as MAP2+ branches originating directly from the soma, following the developers' instructions. After tracing was completed, a text file containing neurite count and length measurement data was generated for each neuron traced and a snapshot of the tracings overlaid on the neuron was saved as a TIFF file. For the analysis, 60 neurons were traced for each line.

Calcium imaging

Neuronal cultures (5 weeks) were incubated with the fluorogenic calcium-sensitive dye Cal-520® AM (10 µM, AAT Bioquest) in neural differentiation medium supplemented with 0.04% Pluronic (P3000MP, ThermoFisher Scientific) at 37 °C for 1 h. At the end of the incubation period, the cultures were rinsed thrice with PBS. Spontaneous calcium activity was acquired at a rate of 50 frames/s with exposure time at 20ms for 3 min using a Nikon CrEST X-Light V3 Spinning Disk microscope with a 25x air objective and NIS Elements software. For analysis, files were sub-stacked using ImageJ software to visualize 1 frame every 2s. 10 ROIs per FOV were defined (90-100 cells per condition in each line) and the mean fluroscence intensity for each ROI across time was exported into an excel sheet. The fluorescence change over time was defined as $\Delta F/F0=(F-F0)/F0$, where F is the fluorescence of a ROI at a specific time point and F0 is the corresponding fluorescence of a background area.

Supplementary References

1 Rao, S. S. *et al.* A 3D map of the human genome at kilobase resolution reveals principles of chromatin looping. *Cell* **159**, 1665-1680 (2014).

2 Kular, L. *et al.* Neuronal methylome reveals CREB-associated neuro-axonal impairment in multiple sclerosis. **11**, 1-20 (2019).

3 Kular, L. *et al.* DNA methylation changes in glial cells of the normal-appearing white matter in Multiple Sclerosis patients. *Epigenetics* **17**, 1311-1330 (2022).

4 Josipović, G. *et al.* Antagonistic and synergistic epigenetic modulation using orthologous CRISPR/dCas9-based modular system. *Nucleic acids research* **47**, 9637-9657 (2019).

5 Kular, L. *et al.* DNA methylation as a mediator of HLA-DRB1*15:01 and a protective variant in multiple sclerosis. *Nat Commun* **9**, 2397, doi:10.1038/s41467-018-04732-5 (2018).

6 Hedström, A. K., Hillert, J., Olsson, T. & Alfredsson, L. Smoking and multiple sclerosis susceptibility. *European journal of epidemiology* **28**, 867-874 (2013).

7 Huynh, J. L. *et al.* Epigenome-wide differences in pathology-free regions of multiple sclerosis-affected brains. *Nat Neurosci* **17**, 121-130, doi:10.1038/nn.3588 (2014).

8 Schirmer, L. *et al.* Neuronal vulnerability and multilineage diversity in multiple sclerosis. *Nature* **573**, 75-82, doi:10.1038/s41586-019-1404-z (2019).

9 Ng, B. *et al.* An xQTL map integrates the genetic architecture of the human brain's transcriptome and epigenome. *Nat Neurosci* **20**, 1418-1426, doi:10.1038/nn.4632 (2017).

10 Aryee, M. J. *et al.* Minfi: a flexible and comprehensive Bioconductor package for the analysis of Infinium DNA methylation microarrays. *Bioinformatics* **30**, 1363-1369, doi:10.1093/bioinformatics/btu049 (2014).

11 Houseman, E. A. *et al.* DNA methylation arrays as surrogate measures of cell mixture distribution. *BMC Bioinformatics* **13**, 86, doi:10.1186/1471-2105-13-86 (2012).

12 Jaffe, A. E. *et al.* Mapping DNA methylation across development, genotype and schizophrenia in the human frontal cortex. *Nature neuroscience* **19**, 40-47 (2016).

13 Andrews, S. V., Ladd-Acosta, C., Feinberg, A. P., Hansen, K. D. & Fallin, M. D. "Gap hunting" to characterize clustered probe signals in Illumina methylation array data. *Epigenetics & chromatin* **9**, 1-21 (2016).

14 Purcell, S. *et al.* PLINK: a tool set for whole-genome association and population-based linkage analyses. *Am J Hum Genet* **81**, 559-575, doi:10.1086/519795 (2007).

15 Kretzschmar, W., Mahajan, A., Sharp, K., McCarthy, M. & Consortium, H. R. A reference panel of 64,976 haplotypes for genotype imputation. *Nature Genetics* **48** (2016).

16 Gabriel, S. B. *et al.* The structure of haplotype blocks in the human genome. *science* **296**, 2225-2229 (2002).

17 Chang, C. C. *et al.* Second-generation PLINK: rising to the challenge of larger and richer datasets. *Gigascience* **4**, s13742-13015-10047-13748 (2015).

18 Vojta, A. *et al.* Repurposing the CRISPR-Cas9 system for targeted DNA methylation. *Nucleic Acids Res* **44**, 5615-5628, doi:10.1093/nar/gkw159 (2016).

19 Concordet, J. P. & Haeussler, M. CRISPOR: intuitive guide selection for CRISPR/Cas9 genome editing experiments and screens. *Nucleic Acids Res* **46**, W242-W245, doi:10.1093/nar/gky354 (2018).

20 Livak, K. J. & Schmittgen, T. D. Analysis of relative gene expression data using real-time quantitative PCR and the 2− ΔΔCT method. *methods* **25**, 402-408 (2001).

21 Ewels, P., Magnusson, M., Lundin, S. & Kaller, M. MultiQC: summarize analysis results for multiple tools and samples in a single report. *Bioinformatics* **32**, 3047-3048, doi:10.1093/bioinformatics/btw354 (2016).
22 Wu, Z., Wang, X. & Zhang, X. Using non-uniform read distribution models to improve isoform expression inference in RNA-Seq. *Bioinformatics* **27**, 502-508, doi:10.1093/bioinformatics/btq696 (2011).
23 Pertea, M. *et al.* StringTie enables improved reconstruction of a transcriptome from RNA-seq reads. *Nat Biotechnol* **33**, 290-295, doi:10.1038/nbt.3122 (2015).
24 Song, W.-M. & Zhang, B. Multiscale embedded gene co-expression network analysis. *PLoS computational biology* **11**, e1004574 (2015).
25 Su, S. *et al.* Glimma: interactive graphics for gene expression analysis. *Bioinformatics* **33**, 2050-2052, doi:10.1093/bioinformatics/btx094 (2017).
26 Whitlock, K. E. & Westerfield, M. The olfactory placodes of the zebrafish form by convergence of cellular fields at the edge of the neural plate. *Development* **127**, 3645-3653 (2000).
27 Xu, R. *et al.* Developing human pluripotent stem cell-based cerebral organoids with a controllable microglia ratio for modeling brain development and pathology. **16**, 1923-1937 (2021).
28 Meijering, E. *et al.* Design and validation of a tool for neurite tracing and analysis in fluorescence microscopy images. **58**, 167-176 (2004).

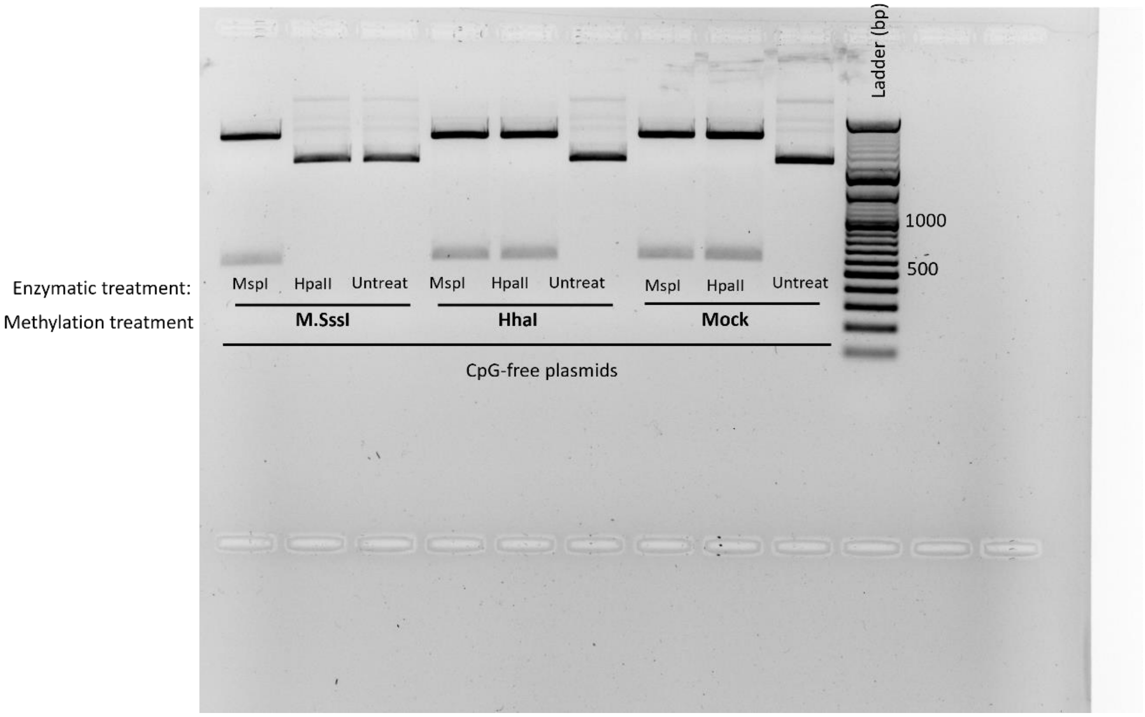

Uncropped scan of the gel used in Supplementary Figure 7.
