# Supplementary material for: A genetic-epigenetic interplay at 1q21.1 locus underlies CHD1L-mediated vulnerability to primary progressive multiple sclerosis
Source: Nat Commun. 2024 Jul 30;15:6419. doi: 10.1038/s41467-024-50794-z (PMC11289459; doi:10.1038/s41467-024-50794-z)
Supplement: Supplementary file 3 — Description of Additional Supplementary Files [file 41467_2024_50794_MOESM3_ESM.pdf]

Description of Additional Supplementary Files

Supplementary Data 1. Description of the cohorts

Supplementary Data 2. Genome-wide methylation analysis (cohort 1)

Supplementary Data 3. Genome-wide meQTL analysis (cohort 1)

Supplementary Data 4. Locus-specific meQTL analysis (cohort 2)

Supplementary Data 5. Brain meQTL data of the SNPs found to control PPMS-specific DMR methylation at 1q21.1 in blood (xQTL serve database)

Supplementary Data 6. Genetic associations (P < 0.05) with PPMS vs BOMS in the Swedish cohorts (SWE, cohort 3). RSID in bold are SNPs with significant P-value after Bonferroni correction.

Supplementary Data 7. Brain meQTLs and eQTLs for all available SNPs associating with PPMS in the SWE and META (SWE + ITA) (GTEx and xQTL serve databases).

Supplementary Data 8: Genetic associations (P < 0.05) with PPMS vs BOMS using meta-analysis of the Swedish (SWE) and Italian (ITA) cohorts with concordant effect. RSID in bold are SNPs with significant P-value after Bonferroni correction.

Supplementary Data 9. Brain correlation network analysis (RNAseq from Huynh et al., 2014 and snRNA-seq from Schirmer et al., 2019). a. Cluster trait Association. b. Modules. c. Correlations

Supplementary Data 10. Gene Ontology analysis (using Ingenuity Pathway Analysis)

Supplementary Data 11. Data sets tested for validation of network modules.

Supplementary Data 12. Primers sequences
