# Supplementary material for: A genetic-epigenetic interplay at 1q21.1 locus underlies CHD1L-mediated vulnerability to primary progressive multiple sclerosis
Source: Nat Commun. 2024 Jul 30;15:6419. doi: 10.1038/s41467-024-50794-z (PMC11289459; doi:10.1038/s41467-024-50794-z)
Supplement: Supplementary file 5 — Reporting Summary [file 41467_2024_50794_MOESM5_ESM.pdf]

nature portfolio

Corresponding author(s): Maja Jagodic
Lara Kular

Last updated by author(s): Jul 1, 2024

Reporting Summary

Nature Portfolio wishes to improve the reproducibility of the work that we publish. This form provides structure for consistency and transparency in reporting. For further information on Nature Portfolio policies, see our Editorial Policies and the Editorial Policy Checklist.

Statistics

For all statistical analyses, confirm that the following items are present in the figure legend, table legend, main text, or Methods section.

n/a	Confirmed	
☐	☒	The exact sample size (*n*) for each experimental group/condition, given as a discrete number and unit of measurement
☐	☒	A statement on whether measurements were taken from distinct samples or whether the same sample was measured repeatedly
☐	☒	The statistical test(s) used AND whether they are one- or two-sided<br>*Only common tests should be described solely by name; describe more complex techniques in the Methods section.*
☐	☒	A description of all covariates tested
☐	☒	A description of any assumptions or corrections, such as tests of normality and adjustment for multiple comparisons
☐	☒	A full description of the statistical parameters including central tendency (e.g. means) or other basic estimates (e.g. regression coefficient) AND variation (e.g. standard deviation) or associated estimates of uncertainty (e.g. confidence intervals)
☒	☐	For null hypothesis testing, the test statistic (e.g. *F*, *t*, *r*) with confidence intervals, effect sizes, degrees of freedom and *P* value noted<br>*Give P values as exact values whenever suitable.*
☒	☐	For Bayesian analysis, information on the choice of priors and Markov chain Monte Carlo settings
☒	☐	For hierarchical and complex designs, identification of the appropriate level for tests and full reporting of outcomes
☐	☒	Estimates of effect sizes (e.g. Cohen's *d*, Pearson's *r*), indicating how they were calculated

Our web collection on statistics for biologists contains articles on many of the points above.

Software and code

Policy information about availability of computer code

Data collection	Softwares: CFX manager (v2.3) for qPCR, ImageJ software, PyroMark Q24 (v2.0.8) for Pyrosequencing
Data analysis	Softwares: GSEA (transciptome analysis) and GraphPad Prism (v9.5.1.733) for statistical analysis.<br>The codes for data analysis used in this study are available at https://gitlab.com/jagodiclab/pahlevan_ppms_chd1l.

For manuscripts utilizing custom algorithms or software that are central to the research but not yet described in published literature, software must be made available to editors and reviewers. We strongly encourage code deposition in a community repository (e.g. GitHub). See the Nature Portfolio guidelines for submitting code & software for further information.

Data

Policy information about availability of data

All manuscripts must include a data availability statement. This statement should provide the following information, where applicable:

- Accession codes, unique identifiers, or web links for publicly available datasets
- A description of any restrictions on data availability
- For clinical datasets or third party data, please ensure that the statement adheres to our policy

The 450K array methylome data generated from whole blood (cohort 1) are available in the Gene Expression Omnibus (GEO) database under accession number GSE106648. The RNA-sequencing data used for correlation network analysis in MS brain are accessible under the accession numbers GSE174647, GSE118257, GSE179427, PRJNA544731 with details presented in Supplementary Data 11. Results from the genetic association analysis (cohort 3) are available in Supplementary

Data. Due to the GDPR regulations, we cannot deposit any personal information including genetic data, which can be shared upon request and under a Data Access Agreement. Source data are provided with this paper.

Research involving human participants, their data, or biological material

Policy information about studies with human participants or human data. See also policy information about sex, gender (identity/presentation), and sexual orientation and race, ethnicity and racism.

Reporting on sex and gender	We did not explore sex-specific MS effects in this study. Self-reported, often validated using molecular data, biological sex was used as a covariate in all analysis.
Reporting on race, ethnicity, or other socially relevant groupings	For the association study and based on the available samples and data we used Swedish and Italian cohorts of MS patients. Please read the Results section under "1q21.1 methylation-controlling variants predispose for PPMS ". More details are available in supplementary files.
Population characteristics	Reported in the Method section in the Supplementary information file.
Recruitment	Reported in the Method section in the Supplementary information file.
Ethics oversight	All experiments on human subjects were approved by the Regional Ethical Review Board in Stockholm and carried out in accordance with institutional guidelines

Note that full information on the approval of the study protocol must also be provided in the manuscript.

Field-specific reporting

Please select the one below that is the best fit for your research. If you are not sure, read the appropriate sections before making your selection.

☒ Life sciences ☐ Behavioural & social sciences ☐ Ecological, evolutionary & environmental sciences

For a reference copy of the document with all sections, see nature.com/documents/nr-reporting-summary-flat.pdf

Life sciences study design

All studies must disclose on these points even when the disclosure is negative.

Sample size	No sample size calculation was performed for the cohorts involved in this study. The sample sizes for all experiments are clearly mentioned in the text.
Data exclusions	We conducted quality control (QC) for all Omics datasets, and no data were excluded from subsequent analysis.
Replication	Number of replicates are specified (1-3 times). No technically successfull experiments were excluded.
Randomization	For all the Omics data, we considered the randomization.
Blinding	Blinding was not applicable for certain analyses in our study, as patients' clinical features needed to be known to accurately perform the relevant analysis. We conducted certain analyses, such as those involving defined cohorts, without blinding. However, for analyses such as "Correlation network analysis in MS brain," we implemented blinding by utilizing published data.

Reporting for specific materials, systems and methods

We require information from authors about some types of materials, experimental systems and methods used in many studies. Here, indicate whether each material, system or method listed is relevant to your study. If you are not sure if a list item applies to your research, read the appropriate section before selecting a response.

Materials & experimental systems

n/a	Involved in the study	
☐	☒	Antibodies
☐	☒	Eukaryotic cell lines
☐	☐	Palaeontology and archaeology
☐	☒	Animals and other organisms
☐	☒	Clinical data
☐	☐	Dual use research of concern
☐	☐	Plants

Methods

n/a	Involved in the study	
☐	☐	ChIP-seq
☐	☒	Flow cytometry
☐	☐	MRI-based neuroimaging

Antibodies

Antibodies used	Please check the supplementary information file, Methods section.
Validation	All the sources of antibodies are clearly mentioned in the manuscript under the "Methods" section. We only used commercially available validated antibodies

Eukaryotic cell lines

Policy information about cell lines and Sex and Gender in Research

Cell line source(s)	Reported in the Method section in the Supplementary information file.
Authentication	None of the cell lines use were authenticated.
Mycoplasma contamination	Mycoplasma contamination was not tested.
Commonly misidentified lines (See ICLAC register)	N/A

Palaeontology and Archaeology

Specimen provenance	N/A
Specimen deposition	N/A
Dating methods	N/A

☐ Tick this box to confirm that the raw and calibrated dates are available in the paper or in Supplementary Information.

Ethics oversight	N/A

Note that full information on the approval of the study protocol must also be provided in the manuscript.

Animals and other research organisms

Policy information about studies involving animals; ARRIVE guidelines recommended for reporting animal research, and Sex and Gender in Research

Laboratory animals	Zebrafish: The wild type AB strain, the chd1lsa14029 (TL) mutant line (#15474), carrying the mutation C>T at the genomic location Chr.6:36844273 (GRCz11), the Tg(olig2:EGFP)vu12 (AB) (#15211) line were obtained from the European Zebrafish Resource Center.Please check the supplementary information file, Methods section.
Wild animals	Our study did not involved wild animals.
Reporting on sex	We did not explore sex-specific MS effects in this study. In all experiments both sexes were used.
Field-collected samples	The study did not involve samples collected from the field
Ethics oversight	All animal experiments were carried out according to the guidelines of the Ethics Committee of IGBMC and ethical approval was obtained from the French Ministry of Higher Education and Research under the number APAFIS#15025-2018041616344504.

Note that full information on the approval of the study protocol must also be provided in the manuscript.

Clinical data

Policy information about clinical studies

All manuscripts should comply with the ICMJE guidelines for publication of clinical research and a completed CONSORT checklist must be included with all submissions.

Clinical trial registration	N/A
Study protocol	N/A
Data collection	N/A
Outcomes	N/A

Dual use research of concern

Policy information about dual use research of concern

Hazards

Could the accidental, deliberate or reckless misuse of agents or technologies generated in the work, or the application of information presented in the manuscript, pose a threat to:

No	Yes	
☒	☐	Public health
☒	☐	National security
☒	☐	Crops and/or livestock
☒	☐	Ecosystems
☒	☐	Any other significant area

Experiments of concern

Does the work involve any of these experiments of concern:

No	Yes	
☒	☐	Demonstrate how to render a vaccine ineffective
☒	☐	Confer resistance to therapeutically useful antibiotics or antiviral agents
☒	☐	Enhance the virulence of a pathogen or render a nonpathogen virulent
☒	☐	Increase transmissibility of a pathogen
☒	☐	Alter the host range of a pathogen
☒	☐	Enable evasion of diagnostic/detection modalities
☒	☐	Enable the weaponization of a biological agent or toxin
☒	☐	Any other potentially harmful combination of experiments and agents

Plants

Seed stocks	N/A
Novel plant genotypes	N/A
Authentication	N/A

ChIP-seq

Data deposition

☐ Confirm that both raw and final processed data have been deposited in a public database such as GEO.

☐ Confirm that you have deposited or provided access to graph files (e.g. BED files) for the called peaks.

Data access links<br>*May remain private before publication.*	N/A
Files in database submission	N/A
Genome browser session<br>(e.g. UCSC)	N/A

Methodology

Replicates	N/A

Sequencing depth	N/A
Antibodies	N/A
Peak calling parameters	N/A
Data quality	N/A
Software	N/A

Flow Cytometry

Plots

Confirm that:

- [ ] The axis labels state the marker and fluorochrome used (e.g. CD4-FITC).
- [ ] The axis scales are clearly visible. Include numbers along axes only for bottom left plot of group (a 'group' is an analysis of identical markers).
- [ ] All plots are contour plots with outliers or pseudocolor plots.
- [ ] A numerical value for number of cells or percentage (with statistics) is provided.

Methodology

Sample preparation	N/A
Instrument	N/A
Software	N/A
Cell population abundance	N/A
Gating strategy	N/A

- [ ] Tick this box to confirm that a figure exemplifying the gating strategy is provided in the Supplementary Information.

Magnetic resonance imaging

Experimental design

Design type	N/A
Design specifications	N/A
Behavioral performance measures	N/A

Acquisition

Imaging type(s)	N/A
Field strength	N/A
Sequence & imaging parameters	N/A
Area of acquisition	N/A

Diffusion MRI ☐ Used ☐ Not used

Preprocessing

Preprocessing software	N/A
Normalization	N/A
Normalization template	N/A

Noise and artifact removal | N/A

Volume censoring | N/A

Statistical modeling & inference

Model type and settings | N/A

Effect(s) tested | N/A

Specify type of analysis: ☐ Whole brain ☐ ROI-based ☐ Both

Statistic type for inference | N/A

(See Eklund et al. 2016)

Correction | N/A

Models & analysis

n/a	Involved in the study
☐	☐ Functional and/or effective connectivity
☐	☐ Graph analysis
☐	☐ Multivariate modeling or predictive analysis

Functional and/or effective connectivity | N/A

Graph analysis | N/A

Multivariate modeling and predictive analysis | N/A
